# Bioactive Phenylalanine Derivatives and Cytochalasins from the Soft Coral-Derived Fungus, *Aspergillus elegans*

**DOI:** 10.3390/md11062054

**Published:** 2013-06-10

**Authors:** Cai-Juan Zheng, Chang-Lun Shao, Lu-Yong Wu, Min Chen, Kai-Ling Wang, Dong-Lin Zhao, Xue-Ping Sun, Guang-Ying Chen, Chang-Yun Wang

**Affiliations:** 1 Key Laboratory of Marine Drugs, Ministry of Education, School of Medicine and Pharmacy, Ocean University of China, Qingdao 266003, China; E-Mails: caijuan2002@163.com (C.-J.Z.); shaochanglun@163.com (C.-L.S.); dieying0719@163.com (M.C.); kailingw@163.com (K.-L.W.); lanseyimi@sina.com (D.-L.Z.); 2 Key Laboratory of Tropical Medicinal Plant Chemistry of Ministry of Education, College of Chemistry and Chemical Engineering, Hainan Normal University, Haikou 571158, China; E-Mail: wuluyong@hainnu.edu.cn; 3 Guangxi Key Laboratory of Marine Biotechnology, Guangxi Institute of Oceanology, Beihai 536000, China; E-Mail: pingty2006@163.com

**Keywords:** soft coral-derived fungus, *Aspergillus elegans*, phenylalanine derivative, cytochalasin, antifouling

## Abstract

One new phenylalanine derivative 4′-OMe-asperphenamate (**1**), along with one known phenylalanine derivative (**2**) and two new cytochalasins, aspochalasin A1 (**3**) and cytochalasin Z24 (**4**), as well as eight known cytochalasin analogues (**5**–**12**) were isolated from the fermentation broth of *Aspergillus elegans* ZJ-2008010, a fungus obtained from a soft coral *Sarcophyton* sp. collected from the South China Sea. Their structures and the relative configurations were elucidated using comprehensive spectroscopic methods. The absolute configuration of **1** was determined by chemical synthesis and Marfey’s method. All isolated metabolites (**1**–**12**) were evaluated for their antifouling and antibacterial activities. Cytochalasins **5**, **6**, **8** and **9** showed strong antifouling activity against the larval settlement of the barnacle *Balanus amphitrite*, with the EC_50_ values ranging from 6.2 to 37 μM. This is the first report of antifouling activity for this class of metabolites. Additionally, **8** exhibited a broad spectrum of antibacterial activity, especially against four pathogenic bacteria *Staphylococcus albus*, *S. aureus*, *Escherichia coli* and *Bacillus cereus*.

## 1. Introduction

Marine-derived fungi have proven to be a promising source of structurally novel and biologically active secondary metabolites that have become interesting and significant resources for drug discovery [1]. Especially, the genus *Aspergillus* has been known to be a major contributor to the bioactive secondary metabolites of marine fungal origin, for example, antibacterial bisabolene-type sesquiterpenoids from sponge-derived fungus, *Aspergillus* sp. [2], cytotoxic hetero-spirocyclic γ-lactams from *A. sydowii* [3], 14-membered macrolides from *A. ostianus* [4], anthraquinone derivatives with naphtho [1,2,3-de] chromene-2,7-dione skeleton from *A. glaucus* [5] and cytochalasins from *A. flavipes* [6].

**Figure 1:**
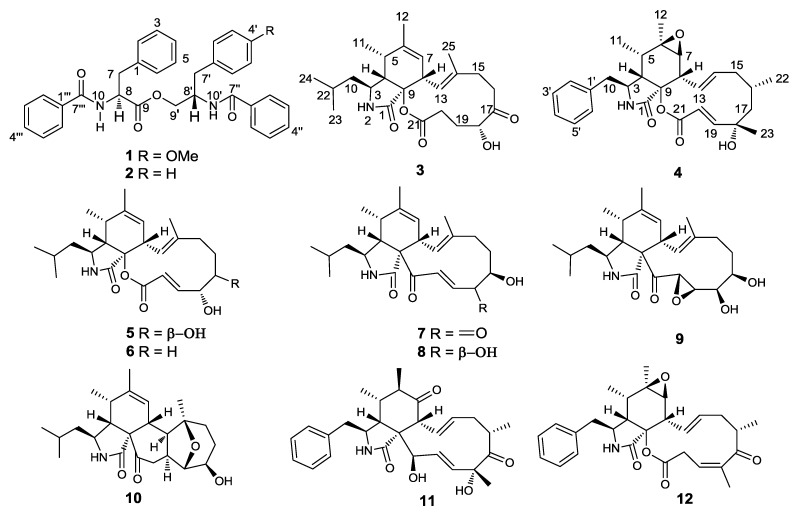
Structures of compounds **1–12**.

In our search for new antibacterial, cytotoxic and antifouling natural products from marine fungi in the South China Sea, we have found several bioactive compounds, including sesquiterpenoids, quinolinone alkaloids, azaphilone derivatives, resorcylic acid lactones and anthraquinone derivatives, from marine fungi through the bioassay-guided isolation [2,7,8,9,10,11,12]. In our ongoing investigations on the marine-derived fungi isolated from marine invertebrates, a fungus, *Aspergillus elegans* ZJ-2008010, obtained from a soft coral, *Sarcophyton* sp., attracted our attention. The EtOAc extract of a fermentation broth of the fungus exhibited antifouling activity against the larval settlement of the barnacle, *Balanus amphitrite*. Bioassay-guided fractionation of the bioactive extract led to the isolation of one new phenylalanine derivative, 4′-OMe-asperphenamate (**1**), together with one known phenylalanine derivative asperphenamate (**2**) [13,14] and two new cytochalasins, aspochalasin A1 (**3**) and cytochalasin Z24 (**4**), as well as eight known cytochalasins (**5**–**12**), determined as aspochalasins I (**5**) and J (**6**) [15], aspochalasins B (**7**) and D (**8**) [16,17], aspochalasin H (**9**) [18], aspergillin PZ (**10**) [19], zygosporin D (**11**) [20] and rosellichalasin (**12**) [21] (Figure 1). All isolated metabolites (**1**–**12**) were evaluated for their antifouling and antibacterial activities. Herein, we report the isolation, structure elucidation and biological activities of these compounds.

## 2. Results and Discussion

**Figure 2:**
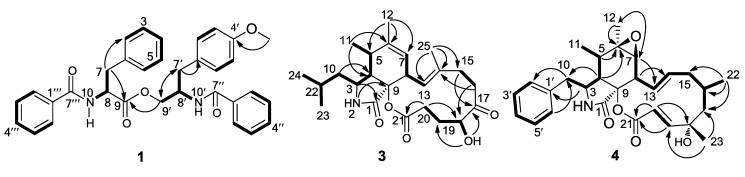
^1^H-^1^H COSY correlations and key heteronuclear multiple bond correlation (HMBC) correlations of compounds **1**, **3** and **4**.

4′-OMe-asperphenamate (**1**) was obtained as a white powder. Its molecular formula was established as C_33_H_32_N_2_O_5_ (19 degrees of unsaturation) from HRESIMS, combined with ^1^H and ^13^C NMR spectroscopic data (Table 1). The ^1^H NMR spectrum of **1** showed the presence of 19 aromatic protons in the downfield, including three monosubstituted phenyls at δ_H_ [7.38 (2H, m), 7.24 (1H, m) and 7.23 (2H, m); 7.65 (2H, m), 7.50 (1H, m) and 7.30 (2H, m); and 7.70 (2H, m), 7.42 (1H, m) and 7.30 (2H, m)] and one disubstituted phenyl at δ_H_ [7.12 (1H, d, *J* = 8.4 Hz), 7.10 (1H, d, *J* = 8.4 Hz), 6.82 (1H, d, *J* = 8.4 Hz) and 6.81 (1H, d, *J* = 8.4 Hz)]. In addition, two secondary amino protons at δ_H_ 6.71 (1H, d, *J* = 8.4 Hz) and 6.59 (1H, d, *J* = 6.6 Hz) were also observed in the downfield. In the highfield, two methine groups at δ_H_ 4.88 (^1^H, m) and 4.62 (1H, m), one oxygenated methylene group at δ_H_ [4.52 (1H, dd, *J* = 10.8, 4.2 Hz) and 4.05 (1H, dd, *J* = 10.8, 3.0 Hz)], one methoxyl group at δ_H_ 3.74 (3H, s), two methylene groups at δ_H_ [3.22 (1H, dd, *J* = 14.4, 6.6 Hz) and 3.16 (1H, dd, *J* = 14.4, 6.6 Hz)] and at δ_H_ [3.00 (1H, dd, *J* = 13.2, 6.6 Hz) and 2.89 (1H, dd, *J* = 13.2, 8.4 Hz)] were observed. These spectroscopic features combined with the ^13^C NMR spectrum suggested that **1** very closely resembled asperphenamate (**2**) [13,14]. The only difference in the ^1^H NMR spectrum was the presence of a singlet methyl signal at δ_H_ 3.74 (3H, s) in **1** instead of an aromatic proton signal at δ_H_ 7.30 (1H, m) in **2**. Furthermore, in the ^13^C NMR spectrum, the C-4′ signal moved downfield significantly [δ_C_ 158.9 (C) in **1** *vs.* 126.8 (CH) in **2**], indicating a methoxy group was located at C-4′. The location of the methoxy group at C-4′ was confirmed by the heteronuclear multiple bond correlation (HMBC) correlation from 4′-OCH_3_ at δ_H_ 3.74 (3H, s) to C-4′ at δ_C_ 158.9 (C). The planar structure of **1** was further confirmed by the ^1^H-^1^H COSY and HMBC experiments (Figure 2). 

**Table 1:** NMR spectroscopic data (600/150 MHz, DMSO-*d*_6_) for compound **1**.

Position	^1^H (*J* in Hz)	^13^C	Position	^1^H (*J* in Hz)	^13^C
1	-	134.2, C	8′	4.62, m	50.4, CH
2	7.23, m	129.4, CH	9′	4.52, dd (10.8, 4.2)	65.4, CH_2_
3	7.38, m	128.8, CH		4.05, dd (10.8, 3.0)	
4	7.24, m	127.2, CH	10′	6.71, d (8.4)	-
5	7.23, m	128.8, CH	1″	-	133.4, C
6	7.38, m	129.4, CH	2″	7.65, m	127.2, CH
7	3.22, dd (14.4, 6.6)	37.4, CH_2_	3″	7.30, m	128.5, CH
	3.16, dd (14.4, 6.6)		4″	7.50, m	132.1, CH
8	4.88, m	55.2, CH	5″	7.30, m	128.5, CH
9	-	172.1, C	6″	7.65, m	126.9, CH
10	6.59, d (6.6)	-	7″	-	167.5, C
1′	-	137.3, C	1′″	-	137.2, C
2′	7.10, d (8.4)	130.3, CH	2′″	7.70, m	127.1, CH
3′	6.81, d (8.4)	114.3, CH	3′″	7.30, m	128.5, CH
4′	-	158.9, C	4′″	7.42, m	131.5, CH
5′	6.82, d (8.4)	114.3, CH	5′″	7.30, m	128.5, CH
6′	7.12, d (8.4)	130.3, CH	6′″	7.70, m	127.1, CH
7′	3.00, dd (13.2, 6.6)	36.7, CH_2_	7′″	-	166.7, C
	2.89, dd (13.2, 8.4)		4′-OCH_3_	3.74, s	54.6, CH_3_

The absolute configurations of C-8 and C-8′ in **1** were determined by chemical synthesis and Marfey’s method [22]. The major acid hydrolysis products of **1** with 6 N HCl at 105 °C for 19 h were Phe and 2-amino-3-(4-methoxyphenyl)-1-propanol. The hydrolysates of **1**, as well as the standard l-Phe and dl-Phe, were derivatized with Marfey’s reagent, 1-fluoro-2,4-dinitrophenyl-5-l-alanine amide (FDAA). Analysis of the FDAA derivatives of the hydrolysates from **1** by HPLC, compared with the FDAA derivatives from l-Phe and dl-Phe, revealed the presence of an l-Phe moiety in **1** (Figure S32). Therefore, the absolute configuration at C-8 was assigned as *S*.

**Figure 3:**
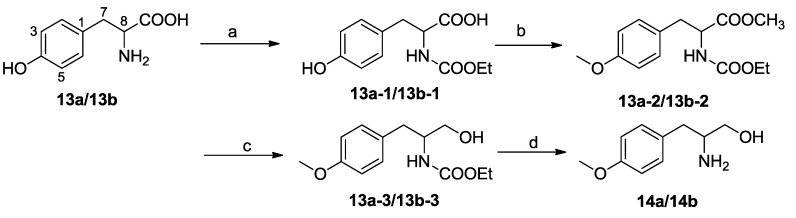
The preparation of compounds **14a** and **14b**.

To determine the absolute configuration at C-8′, (*R*/*S*)-2-amino-3-(4-methoxyphenyl)-1-propanol (**14a**) and (*S*)-2-amino-3-(4-methoxyphenyl)-1-propanol (**14b**) were synthesized. Compounds **14a** and **14b** were prepared from dl-Tyr (**13a**) and l-Tyr (**13b**) (Figure 3), respectively, according to the literature method [23]. Also, **14a** and **14b** were derivatized with Marfey’s reagent (FDAA). Analysis of the FDAA derivatives of the hydrolysates from **1** by HPLC, compared with the FDAA derivatives from **14a** and **14b**, revealed the presence of a **14b** moiety in **1** (Figure S32). Accordingly, the absolute configuration at C-8′ was assigned as *S*. The 8*S*8′*S* configurations in **1** were consistent with those in asperphenamate (**2**), confirming that these two compounds share the same biogenetic path way.

Aspochalasin A1 (**3**) was isolated as a white powder. Its molecular formula was established as C_24_H_35_NO_5_ (eight degrees of unsaturation) by HRESIMS, combined with ^1^H and ^13^C NMR spectroscopic data (Table 2). The ^1^H NMR spectrum recorded in CDCl_3_ intuitively revealed five methyl groups, two olefinic protons [δ_H_ 6.00, 1H, d, *J* = 11.4 Hz, H-13; δ_H_ 5.37, 1H, br s, H-7], an amide group (δ_H_ 5.93, 1H, br s, NH), six methine protons, five methylene units, along with one hydroxy group (Table 2). These features characteristically revealed the structure of **3** as possessing a (2-methylpropyl) isoindolone moiety, consistent with a cytochalasin skeleton [16]. These structural features were also confirmed by the ^13^C NMR and DEPT spectra (Table 2). In the ^13^C NMR spectrum, except for the carbon signals of the (2-methylpropyl) isoindolone moiety, there were also ten resonance signals revealed that **3** possessed a 12-membered lactone macrocycle, containing a methyl group (δ_C_ 15.9, CH_3_, C-25), a double bond [δ_C_ 125.6 (CH), C-13 and δ_C_ 136.2 (C), C-14] and two carbonyl groups [δ_C_ 212.7 (C), C-17 and δ_C_ 171.8 (C), C-21]. The ^1^H and ^13^C NMR spectra of **3** were closely related to those of aspochalasin M [24]. The obvious difference was the absence of the macrocyclic ketone moiety as in aspochalasin M at δ_C_ 214.5 (C-21) and the presence of a lactone at δ_C_ 171.8 (C-21) in **3**. The differences were also observed for the chemical shifts of H-18/C-18 [δ_H_ 5.00 (1H, ddd, *J* = 10.8, 3.6, 3.6 Hz) and δ_C_ 74.0 (CH) in **3** *vs.* δ_H_ 4.15 (1H, dd, *J* = 5.0, 5.0 Hz) and δ_C_ 77.4 (CH) in aspochalasin M] and the large coupling constant *J* = 10.8 Hz between H-18 (δ_H_ 5.00) and H-19 (δ_H_ 1.53) in **3**, instead of the small coupling constant *J* = 5.0 Hz between H-18 (δ_H_ 4.15) and H-19 (δ_H_ 2.00) in aspochalasin M. These features established an axial H-18 in **3** in contrast to an equatorial H-18 in aspochalasin M. This suggested that the OH at C-18 in **3** has a α-orientation different from the β-orientation in aspochalasin M. The ^1^H-^1^H COSY and HMBC allowed the deduction of the gross structure of **3** as shown in Figure 2.

**Table 2:** NMR spectroscopic data (600/150 MHz, CDCl_3_) for compounds **3** and **4**.

Position	3		4	
	^1^H (*J* in Hz)	^13^C	^1^H (*J* in Hz)	^13^C
1	-	172.0, C	-	166.9, C
2	5.93, br s	-	6.30, br s	-
3	3.00, m	52.6, CH	3.68, m	53.6, CH
4	2.49, dd (4.2, 3.6)	52.6, CH	3.04, br d (5.4)	49.2, CH
5	2.74, m	35.1, CH	2.22, m	35.9, CH
6	-	141.2, C	-	57.2, C
7	5.37, br s	122.5, CH	2.74, d (5.4)	60.8, CH
8	3.43, d (10.8)	42.1, CH	2.99, d (10.8, 5.4)	47.0, CH
9	-	86.8, C	-	85.2, C
10	1.96, m	46.4, CH_2_	2.86, dd (12.6, 8.4)	45.1, CH_2_
	1.57, m		2.77, dd (12.6, 6.6)	
11	1.21, d (6.6)	14.3, CH_3_	0.93, d (6.6)	12.7, CH_3_
12	1.78, br s	20.3, CH_3_	1.17, br s	19.5, CH_3_
13	6.00, d (10.8)	125.6, CH	6.03, dd (13.2, 10.8)	124.2, CH
14	-	136.2, C	5.21, ddd (13.2, 10.8, 3.6)	138.8, CH
15	2.68, m	37.2, CH_2_	2.11, m	44.1, CH_2_
	2.39, m		2.08, m	
16	2.69, m	35.5, CH_2_	1.27, m	29.3, CH
	2.45, m			
17	-	212.7, C	1.76, dd (13.2, 1.8)	53.8, CH_2_
			1.68, dd (13.2, 5.4)	
18	5.00, ddd (10.8, 3.6, 3.6)	74.0, CH	-	72.8, C
19	2.10, m	29.1, CH_2_	7.11, d (15.6)	159.1, CH
	1.53, m			
20	2.65, m	31.4, CH_2_	5.69, d (15.6)	119.1, CH
	2.44, m			
21	-	171.8, C	-	172.8, C
22	1.19, m	25.6, CH	1.03, d (6.6)	27.1, CH_3_
23	0.93, d (6.6)	21.0, CH_3_	1.33, s	22.0, CH_3_
24	0.90, d (6.6)	23.9, CH_3_	-	-
25	1.80, br s	15.9, CH_3_	-	-
1′	-	-	-	137.0, C
2′/6′	-	-	7.15, br d (7.2)	129.4, CH
3′/5′	-	-	7.30, m	129.0, CH
4′	-	-	7.25, m	127.1, CH
18-OH	3.56, d (3.6)			

**Figure 4:**
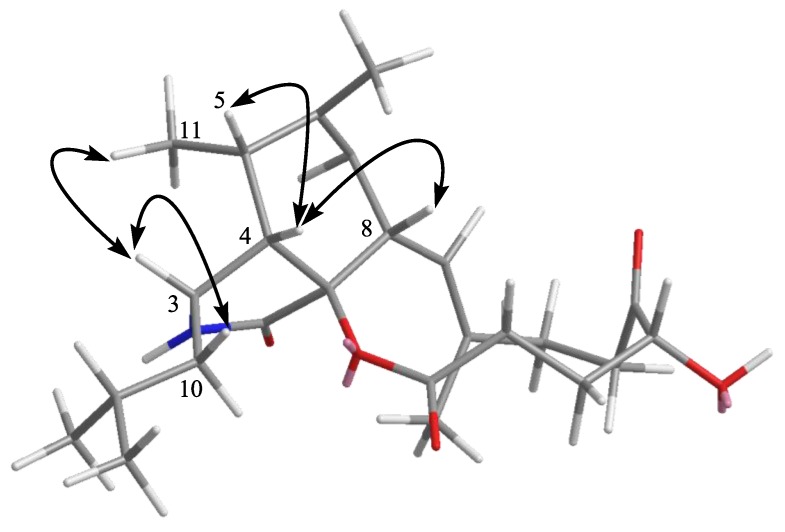
Key NOESY correlations of compound **3**.

The relative stereochemistry of **3** was determined by NOESY experiments and comparison of NMR data with those of aspochalasin M [24]. In the NOESY experiments (Figure 4), the signal of H-3 showed correlations with H-10 and H-11 and H-4 with H-5 and H-8, indicating that the relative configurations of the perhydroisoindol-1-one moiety in **3** were in accord with those of reported cytochalasins. The modified Mosher’s method [25] was tried to determine the absolute configuration of C-18 in **3**; unfortunately, the reactions failed. A literature search revealed that the stereochemistry of the cyclohexane and isoindole moieties in all isolated cytochalasins, so far, are the same [26]. Therefore, based on the above data and the biogenesis consideration, **3** was determined as aspochalasin A1. 

Cytochalasin Z24 (**4**) was also obtained as a white power. Its molecular formula was established as C_28_H_35_NO_5_ by HRESIMS. The general features of its NMR spectroscopic data (Table 2) were markedly similar to those of cytochalasin Z22 [27]. Detailed comparison of NMR data of these two compounds suggested that they had the same 10-phenyl-substituted 6,7-epoxyperhydroisoindol-1-one skeleton. The only significant difference in the ^1^H NMR spectrum was the presence of a methylene group at δ_H_ [1.76 (dd, *J* = 13.2, 1.8 Hz) and 1.68 (dd, *J* = 13.2, 5.4 Hz)] for H-17 in **4**, and in the ^13^C-NMR spectrum, a methylene group at δ_C_ 53.8 (CH_2_) for C-17 was observed in **4**, instead of a carbonyl carbon at δ_C_ 212.3 (C) for C-17 in cytochalasin Z22. The gross structure of **4** was further confirmed by ^1^H-^1^H COSY and HMBC spectra (Figure 2).

The configuration of the core skeleton, 10-phenyl-substituted 6,7-epoxyperhydroisoindol-1-one in **4** could be assigned based on biogenetic origin with those of cytochalasin Z22 [27]. In the ^1^H and ^13^C NMR spectra of **4**, the chemical shifts and coupling constants of the H-atoms of C-3, C-4, C-5, C-7, C-8, 11-Me and 12-Me and the chemical shifts of the C-atoms of C-3–C-8, 11-Me and 12-Me were similar to those of cytochalasin Z22, suggesting that C-3–C-8 in **4** possess the same configurations as in Z22. The configurations of C-16 and C-18 of the macrocyclic moiety in **4** were assigned by comparison of the NMR data with those of [11]-cytochalasa-6(12),13,19-triene-1,21-dione-7,18-dihydroxy-16,18-dimethyl-10-phenyl-(7S*,13*E*,16S*,18S*,19*E*) [28], [δ_H_ 1.27 (1H, m) and δ_C_ 29.3 (CH) for C-16, δ_C_ 72.8 (C) for C-18 in **4**] *vs.* [δ_H_ 1.33 (^1^H, m) and δ_C_ 28.8 (CH) for C-16, δ_C_ 72.8 (C) for C-18 in [11]-cytochalasa-6(12),13,19-triene-1,21-dione-7,18-dihydroxy-16,18-dimethyl-10-phenyl-(7S*,13*E*,16S*,18S*,19*E*)]. These results indicated that the two compounds shared the same macrocyclic moiety, and these were also confirmed by other known cytochalasins based on the shared biogenesis. The absolute configuration of **4** was then deduced as 3*S*,4*S*,5*S*,6*R*,7*S*,8*S*,9*S*,13*E*,16*S*,18*S*,19*E*. Therefore, the structure of **4** was established and named cytochalasin Z24. 

The structures of known compounds (**2**, **5**–**12**) were identified by comparison of their spectroscopic data with those in the literature [13,14,15,16,17,18,19,20,21]. Among them, asperphenamate (**2**), an uncommon phenylalanine derivative, has already been isolated from some bioactive natural sources, such as fungi, *Aspergillus flavipes* [29], *Penicillium megasporum* [30], *P. brevicompactum* [31] and *P. canadense* [32], and plants, *Anaphalis subumbellata* [33], *Artemisia anomala* [34] and *Croton hieronymi* [13]. In the present paper, this is the first report of isolated asperphenamate from marine-derived fungus*.*

Cytochalasins are a large group of fungal alkaloids with a wide range of biological activities targeting cytoskeletal processes [35]. Cytochalasin biosynthesis has been revealed by the formation of an acetate- and methionine-derived octa- or nonaketide chain and the attachment of an amino acid. The type of cytochalasins depends on the incorporated amino acids as structural subunits [36,37]. Compounds **3**–**9**, **11** and **12** are a class of cytochalasins with 12-membered or 11-membered carbocyclic (or oxygen-containing) rings connecting the C-8 and C-9 positions of a perhydroisoindol-1-one moiety. The substituents at C-3 in compounds **3** and **5**–**9** is a 2-methylpropyl group; and in compounds **4**, **11** and **12** is a phenyl group, belonging to the class of 10-phenyl-[11]-cytochalasin. Compound **10** belongs to an unusual type of aspochalasins with a pentacyclic system, of which only three examples have been reported.

All the isolated compounds were evaluated for antifouling activity against the larval settlement of the barnacle, *Balanus amphitrite*. Compounds **5**, **6**, **8** and **9** showed strong antifouling activity with the EC_50_ values of 34, 14, 6.2 and 37 μM, respectively. Despite the slight structural differences in their macrocycles, **8**, bearing an α,β-unsaturated ketone moiety, was found to be considerably more active than **6**, with an α,β-unsaturated lactone moiety, suggesting the importance of an electrophilic α,β-unsaturated carbonyl moiety for the antifouling activity of these cytochalasins. Compound **8** displayed more activity than **9**, indicating that the double-bond at C-19 and C-20 might be essential for the antifouling activity of cytochalasins. This is the first report of antifouling activity for this class of metabolites. 

The antibacterial activity of all isolated compounds were also assessed against six terrestrialpathogenic bacteria and two marine pathogenic bacteria. Compounds **1**, **2**, **5**, **8** and **10** exhibited selective antibacterial activity (Table 3). Compounds **1** and **2** showed selective antibacterial activity against *S. epidermidis*, with the MIC values of 10 μM for each. Compound **5** showed moderate activity against *S. epidermidis* and *S. aureus* with the MIC values of 20 and 10 μM, respectively, while **8** showed a broad spectrum of antibacterial activity, especially towards four pathogenic bacteria, *S. epidermidis*, *S. aureus*, *E. coli* and *B. cereus*. 

**Table 3:** Antibacterial activity for compounds **1**, **2**, **5**, **8** and **10**.

Compound	MIC (μM)			
	*S. epidermidis*	*S. aureus*	*E. coli*	*B. cereus*
**1**	10	>20	>20	>20
**2**	10	>20	>20	>20
**5**	20	10	>20	>20
**8**	10	10	10	10
**10**	20	>20	>20	>20
Ciprofloxacin ^a^	0.30	0.30	0.60	1.20

^a^ Ciprofloxacin was used as a positive control.

## 3. Materials and Methods

### 3.1. General Experimental Procedures

Optical rotations were measured on a JASCO P-1020 digital polarimeter. IR spectra were recorded on a Nicolet Nexus 470 spectrophotometer. ^1^H and ^13^C NMR spectra were recorded on a JEOL Eclips-600 spectrometer at 600 MHz for ^1^H and 150 MHz for ^13^C in DMSO-*d*_6_ or CDCl_3_. Chemical shifts δ are reported in ppm, using TMS as internal standard, and coupling constants (*J*) are in Hz. ESIMS and HRESIMS spectra were measured on a Q-TOF Ultima Global GAA076 LC mass spectrometer. HPLC separation was performed using a Hitachi L-2000 prep-HPLC system coupled with a Hitachi L-2455 photodiode array detector. A Kromasil C18 preparative HPLC column (10 × 250 mm, 5 μm and 4.6 × 250 mm, 5 μm) was used. Analysis of FDAA derivatives by HPLC was performed using Waters 2695 prep-HPLC system coupled with a Waters 2489 UV detector. A Waters C18 analytical HPLC column (4.6 × 250 mm, 5 μm) was used. Silica gel (Qing Dao Hai Yang Chemical Group Co.; Qing Dao, China; 200–300 mesh), octadecylsilyl silica gel (Unicorn, Merck KGaA, Darmstadt, Germany; 45–60 μm) and Sephadex LH-20 (Amersham Biosciences Inc., Piscataway, NJ, USA) were used for column chromatography (CC). Precoated silica gel plates (Yan Tai Zi Fu Chemical Group Co.; Yan Tai, China; G60, F-254) were used for thin layer chromatography (TLC). 

### 3.2. Fungal Materials

The fungal strain, *Aspergillus elegans* ZJ-2008010, was isolated from a piece of tissue from the inner part of the fresh soft coral, *Sarcophyton* sp. (GX-WZ-20080011), which was collected from the Weizhou coral reef in the South China Sea in September, 2008. The strain was deposited in the Key Laboratory of Marine Drugs, the Ministry of Education of China, School of Medicine and Pharmacy, Ocean University of China, Qingdao, China, with the access code ZJ-2008010. The fungal strain was cultivated in 50 L potato glucose liquid medium (15 g of glucose and 30 g of sea salt in 1 L of potato infusion, in 1 L Erlenmeyer flasks, each containing 300 mL of culture broth) at 25 °C without shaking for 4 weeks. 

### 3.3. Identification of Fungus

The Fungus was identified according to its morphological characteristics and a molecular biological protocol by 16S rRNA amplification and sequencing of the ITS region. The sequence data have been submitted to GenBank, with an accession number JF694928, and the fungal strain was identified as *Aspergillus elegans*.

### 3.4. Extraction and Isolation

The fungal cultures were filtered through cheesecloth, and the filtrate was extracted with EtOAc (3 × 50 L, 12 h each). The organic extracts were concentrated *in vacuo* to yield an oily residue (20.2 g), which was subjected to silica gel column chromatography (CC) (petroleum ether, EtOAc v/v, gradient 100:0–0:100) to generate nine fractions (Fr. 1–Fr. 9). Fr. 2 was isolated by CC on silica gel eluted with petroleum ether-EtOAc (4:1) and then subjected to Sephadex LH-20 CC eluting with mixtures of petroleum ether-CHCl3-MeOH (2:1:1) to obtain compounds **1** (3.0 mg) and **2** (20.0 mg). Fr. 3 was subjected to repeated Sephadex LH-20 CC (CHCl_3_/MeOH, v/v, 1:1) and further purified by using HPLC on an ODS semi-preparative column (Kromasil C_18_, 10 × 250 mm, 5 μm, 2 mL/min) eluted with 85% MeOH/H_2_O to obtain compounds **3** (3.6 mg), **4** (4.0 mg), **7** (10.0 mg), **11** (7.0 mg) and **12** (5.0 mg). Fr. 4 was subjected to repeated Sephadex LH-20 CC (CHCl3/MeOH, v/v, 1:1) and further purified on HPLC (82% MeOH/H_2_O) to afford compounds **5** (10.8 mg), **6** (8.2 mg) and **8** (11.0 mg). Fr. 5 was subjected to repeated Sephadex LH-20 CC (MeOH) and further purified on HPLC (60% MeOH/H_2_O) to afford compounds **9** (9.0 mg) and **10** (12.0 mg). 

4′-OMe-asperphenamate (**1**): White powder; [α]^24^_D_ −38.0 (*c* 0.20, MeOH); UV (MeOH) λ_max_ (log ε) 214(2.90), 280(2.50), 314(1.20) nm; IR (KBr) ν_max_ 3448, 1712, 1624 cm^−1^; ^1^H and ^13^C NMR: see Table 1; HRESIMS *m*/*z* 537.2215 [M + H]^+^ (calcd. for C_33_H_33_N_2_O_5_, 537.2209). 

Aspochalasin A1 (**3**): White powder; [α]^24^_D_ +12.0 (*c* 0.15, CHCl3); UV (MeOH) λ_max_ (log ε) 214(2.90); IR (KBr) ν_max_ 3462, 1700, 1448 cm^−1^; ^1^H and ^13^C NMR: see Table 2; HRESIMS *m*/*z* 418.2582 [M + H]^+^ (calcd. for C_24_H_36_NO_5_, 418.2593). 

Cytochalasin Z24 (**4**): White powder; [α]^24^_D_ −25.0 (*c* 0.12, CHCl3); UV (MeOH) λ_max_ (log ε) 214(4.1), 232(3.1), 265(3.0), 289(2.9); IR (KBr) νmax 3310, 1710, 1446 cm^−1^; ^1^H and ^13^C NMR: see Table 2; HRESIMS *m*/*z* 466.2407 [M + H]^+^ (calcd. for C_28_H_36_NO_5_, 466.2410). 

### 3.5. Synthesis of Compounds **14a** and **14b**

**(*S*/*R*)-3-(4-Hydroxyphenyl)-2-[(ethoxycarbonyl)amino] propionic acid (13a-1) or (*S*)-3-(4-hydroxyphenyl)-2-[(ethoxycarbonyl)amino] propionic acid (13b-1)**: Ethyl chloroformate (255 μL, 3.3 mmol) was added to a solution of dl-Tyr or l-Tyr (**13a** or **13b **, 543 mg, 3 mmol, respectively) and NaHCO_3_ (750 mg, 9 mmol, respectively) in a mixture of H_2_O/THF (15 mL/15 mL, respectively). After stirring at room temperature (rt) overnight, the mixture was diluted with H_2_O and washed with Et_2_O, successively. The aqueous layer was acidified and extracted with EtOAc (3 × 18 mL). The combined extracts were washed (H_2_O), dried (Na_2_SO_4_) and concentrated to afford 500 mg (92%) of **13a-1** or **13b-1**, respectively, colorless oil.

**Methyl (*S*/*R*)-2-[(ethoxycarbonyl)amino]-3-(4-methoxyphenyl) propanoate (13a-2) or methyl (*S*)-2-[(ethoxycarbonyl)amino]-3-(4-methoxyphenyl) propanoate (13b-2)**: CH_3_I (370 μL, 6 mmol) was added to a solution of **13a-1** or **13b-1** (500 mg, 1.97 mmol, respectively) in dry acetone (5 mL, respectively) containing suspended K_2_CO_3_ (822 mg, 6 mmol, respectively). The mixture was refluxed at rt overnight, then diluted with H_2_O and extracted with EtOAc (3 × 50 mL, respectively). The combined extracts were washed (H_2_O), dried (Na_2_SO_4_) and concentrated, afforded 410 mg (82%) of **13a-2** or **13b-2**, respectively, colorless oil. 

**(*R*/*S*)-2-[(Ethoxycarbonyl)amino]-3-(4-methoxyphenyl) propan-1-ol (13a-3) and (*S*)-2-[(ethoxycarbonyl)amino]-3-(4-methoxyphenyl) propan-1-ol (13b-3)**: A solution of **13a-2** or **13b-2** (200 mg, 0.71 mmol, respectively) in THF (10 mL, respectively) was added dropwise over 2 h to a cold (0 °C) solution of LiAlH_4_ (48 mg, 1.26 mmol, respectively) in THF (10 mL, respectively) maintained under Ar. The mixture was stirred overnight at rt; then, it was cooled to 0 °C and extracted with EtOAc. The combined extracts were washed (H_2_O), dried (Na_2_SO_4_) and concentrated. The crude product over silica gel (petroleum ether/EtOAc: 4/1) afforded 160 mg (80%) of **13a-3** or **13b-3**, respectively, colorless oil. 

**(*S*/*R*)-2-Amino-3-(4-methoxyphenyl)-1-propanol (14a) and (*S*)-2-amino-3-(4-methoxyphenyl)-1-propanol (14b)**: A solution of **13a-3** or **13b-3** (160 mg, 0.63 mmol) and KOH (900 mg, 16.3 mmol, respectively) in CH3OH/H_2_O (2:1, 7 mL, respectively) was stirred at 80 °C 4 h. The mixture was then extracted with EtOAc (3 × 50 mL). The combined extracts were washed (H_2_O), dried (Na_2_SO_4_) and concentrated. The crude product was over silica gel (petroleum ether/EtOAc: 3/1) to yield 120 g (75%) of **14a** or **14b**, respectively, white solid.

### 3.6. Absolute Configuration Determination of **1** by Marfey’s Method [22]

A solution of **1** (1.5 mg) in 6 M HCl (1 mL) was heated to 105 °C for 19 h. The solution was then evaporated to dryness and the residue redissolved in H_2_O (250 μL). A 50 μL portion of the acid hydrolysate solution was then placed in a 1 mL reaction vial and treated with a 1% solution of FDAA (200 μL) in acetone followed by 1.0 M NaHCO_3_ (40 μL). The reaction mixture was heated at 45 °C for 1 h, cooled to room temperature and then acidified with 2.0 M HCl (20 μL). In a similar fashion, **14a** and **14b** and the standard dl- and l-Phe were derivatized with FDAA separately. The FDAA derivatives of the hydrolysates, **14a** and **14b** and standard amino acids were subjected to HPLC analysis (Waters C18 column; 5 μm, 4.6 × 250 mm; 1.0 mL/min) at 30 °C using the following gradient program: solvent A, H_2_O + 0.1% TFA; solvent B, MeCN; linear gradient: 0 min, 25% B, 40 min, 60% B, 45 min, 100% B; UV detection at 340 nm. The retention times for the FDAA derivatives of hydrolysates of **1** were 22.3 and 23.6 min, respectively; standard l-Phe, d-Phe, (*S*)-2-amino-3-(4-methoxyphenyl)-1-propanol (**14b**) and (*R*)-2-amino-3-(4-methoxyphenyl)-1-propanol were 23.6, 25.7, 22.3 and 24.1 min, respectively (Figure S28, Supporting Information).

### 3.7. Biological Assays

Antifouling activity against the larval-attachment was determined using cyprids of the barnacle, *B. amphitrite* Darwin, according to literature procedures [38]. Adults of *B. amphitrite* exposed to air for more than 6 h were collected from the intertidal zone in Hong Kong and then were placed in a container filled with 0.22 μm of filtered seawater (FSW) to release nauplii. The collected nauplii were reared to the cyprid stage according to the method described by Thiyagarajan *et al*. [38]. When kept at 26–28 °C and fed with *Chaetoceros gracilis*, larvae developed to cyprids on the fourth day. Fresh cyprids were used in the tests. Larval settlement assays were performed using 24-well polystyrene plates (Becton Dickinson 353047 [39]). 

Antibacterial activity was determined against six terrestrial pathogenic bacteria, including *Staphylococcus epidermidis*, *S. aureus*, *Escherichia coli*, *Bacillus subtilis*, *B. cereus* and *Micrococcus luteus*, and two marine pathogenic bacteria, *Vibrio parahaemolyticus* and *Listonella anguillarum*, by the microplate assay method [40]. 

## 4. Conclusions

Twelve secondary metabolites, including two phenylalanine derivatives (**1**,**2**) and ten cytochalasins (**3**–**12**), have been isolated from the fermentation broth of a soft coral-derived fungus, *Aspergillus elegans* ZJ-2008010. Compound **1** is a new phenylalanine derivative, and **3** and **4** are new cytochalasin analogues. Their structures and the relative configurations were elucidated using comprehensive spectroscopic methods. The absolute configuration of **1** was determined by chemical synthesis and Marfey’s method. Asperphenamate (**2**) is the first report of isolated asperphenamate from marine-derived fungus*.* Compound **10** belongs to an unusual type of aspochalasins with a pentacyclic system, of which only three examples have been reported. Cytochalasins showed strong antifouling activity against the larval settlement of the barnacle, *B. amphitrite*. This is the first report of antifouling activity for this class of metabolites.

## Supplementary Files

Supplementary File 1 Supplementary (PDF, 1846 KB)
