# Supplementary material for: Bioactive Phenylalanine Derivatives and Cytochalasins from the Soft Coral-Derived Fungus, Aspergillus elegans
Source: Mar Drugs. 2013 Jun 10;11(6):2054–68. doi: 10.3390/md11062054 (PMC3721221; doi:10.3390/md11062054)
Supplement: Supplementary File 1 — Supplementary (PDF, 1846 KB) [file marinedrugs-11-02054-s001.pdf]

Supporting Information

Figure S1. ^{1}H NMR (600 MHz, DMSO-d_6) spectrum of compound **1**
Figure S2. ^{13}C NMR (150 MHz, DMSO-d_6) spectrum of compound **1**
Figure S3. HMQC (DMSO-d_6) spectrum of compound **1**
Figure S4. ^{1}H-^{1}H COSY (DMSO-d_6) spectrum of compound **1**
Figure S5. HMBC spectrum (DMSO-d_6) of compound **1**
Figure S6. HRESIMS spectrum of compound **1**
Figure S7. ^{1}H NMR (600 MHz, $CDCl_3$) spectrum of compound **3**
Figure S8. ^{13}C NMR (150 MHz, $CDCl_3$) spectrum of compound **3**
Figure S9. HMQC ($CDCl_3$) spectrum of compound **3**
Figure S10. ^{1}H-^{1}H COSY ($CDCl_3$) spectrum of compound **3**
Figure S11. HMBC ($CDCl_3$) spectrum of compound **3**
Figure S12. NOESY ($CDCl_3$) spectrum of compound **3**
Figure S13. HRESIMS spectrum of compound **3**
Figure S14. ^{1}H NMR (600 MHz, $CDCl_3$) spectrum of compound **4**
Figure S15. ^{13}C NMR (150 MHz, $CDCl_3$) spectrum of compound **4**
Figure S16. HMQC ($CDCl_3$) spectrum of compound **4**
Figure S17. ^{1}H-^{1}H COSY ($CDCl_3$) spectrum of compound **4**
Figure S18. HMBC ($CDCl_3$) spectrum of compound **4**
Figure S19. HRESIMS spectrum of compound **4**
Figure S20. ^{1}H NMR (400 MHz, Acetone-d_6) spectrum of compounds **13a-1/13b-1**
Figure S21. ^{13}C NMR (100 MHz, Acetone-d_6) spectrum of compounds **13a-1/13b-1**
Figure S22. ESIMS spectrum of compounds **13a-1/13b-1**
Figure S23. ^{1}H NMR (400 MHz, Acetone-d_6) spectrum of compounds **13a-2/13b-2**
Figure S24. ^{13}C NMR (100 MHz, Acetone-d_6) spectrum of compounds **13a-2/13b-2**
Figure S25. ESIMS spectrum of compounds **13a-2/13b-2**
Figure S26. ^{1}H NMR (400 MHz, Acetone-d_6) spectrum of compounds **13a-3/13b-3**
Figure S27. ^{13}C NMR (100 MHz, Acetone-d_6) spectrum of compounds **13a-3/13b-3**
Figure S28. ESIMS spectrum of compounds **13a-3/13b-3**
Figure S29. ^{1}H NMR (400 MHz, Acetone-d_6) spectrum of compounds **14a/14b**
Figure S30. ^{13}C NMR (100 MHz, Acetone-d_6) spectrum of compounds **14a/14b**
Figure S31. ESIMS spectrum of compounds **14a/14b**
Figure S32. The determination of the absolute configuration of **1** by Marfey's Method
S1. The spectroscopic data of **13a-1/13b-1**, **13a-2/13b-2**, **13a-3/13b-3**, **14a/14b**

Figure S1. ^{1}H NMR (600 MHz, DMSO-d_6) spectrum of compound **1**.

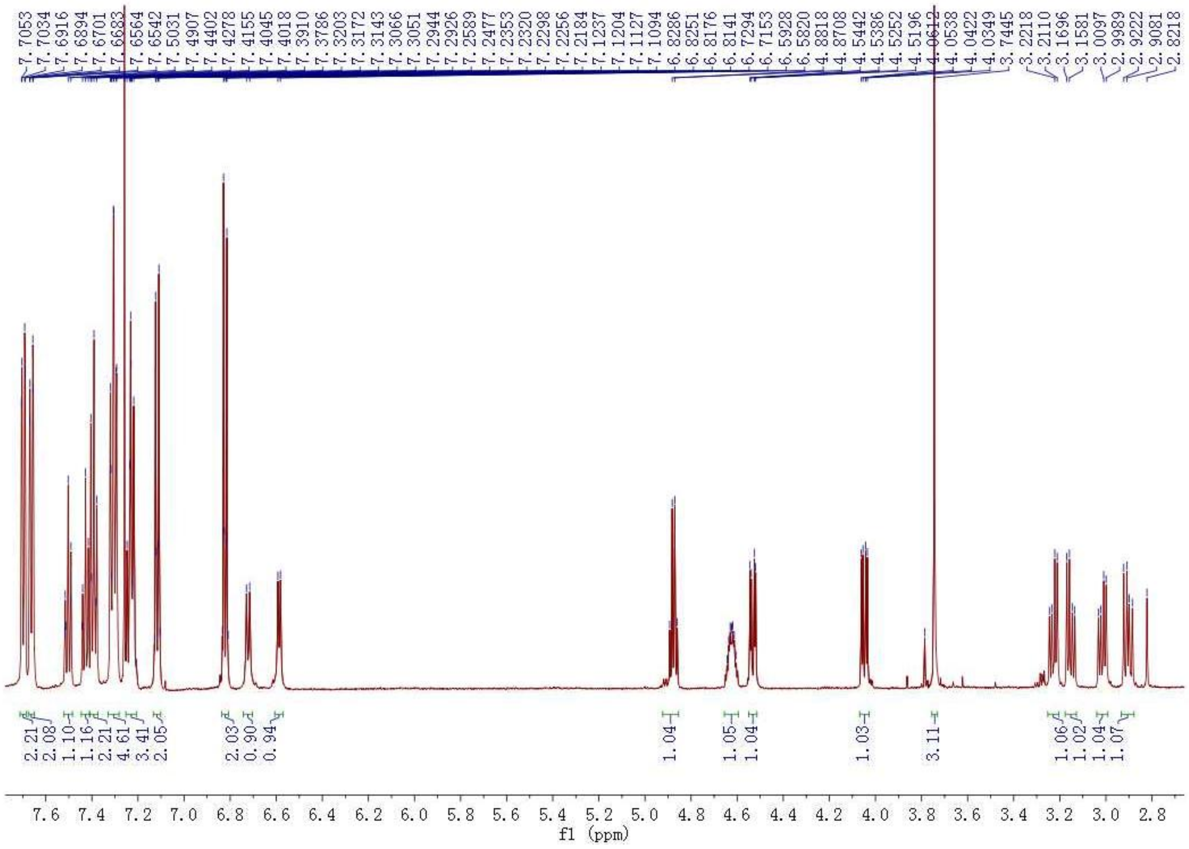

Figure S2. ^{13}C NMR (150 MHz, DMSO-d_6) spectrum of compound **1**.

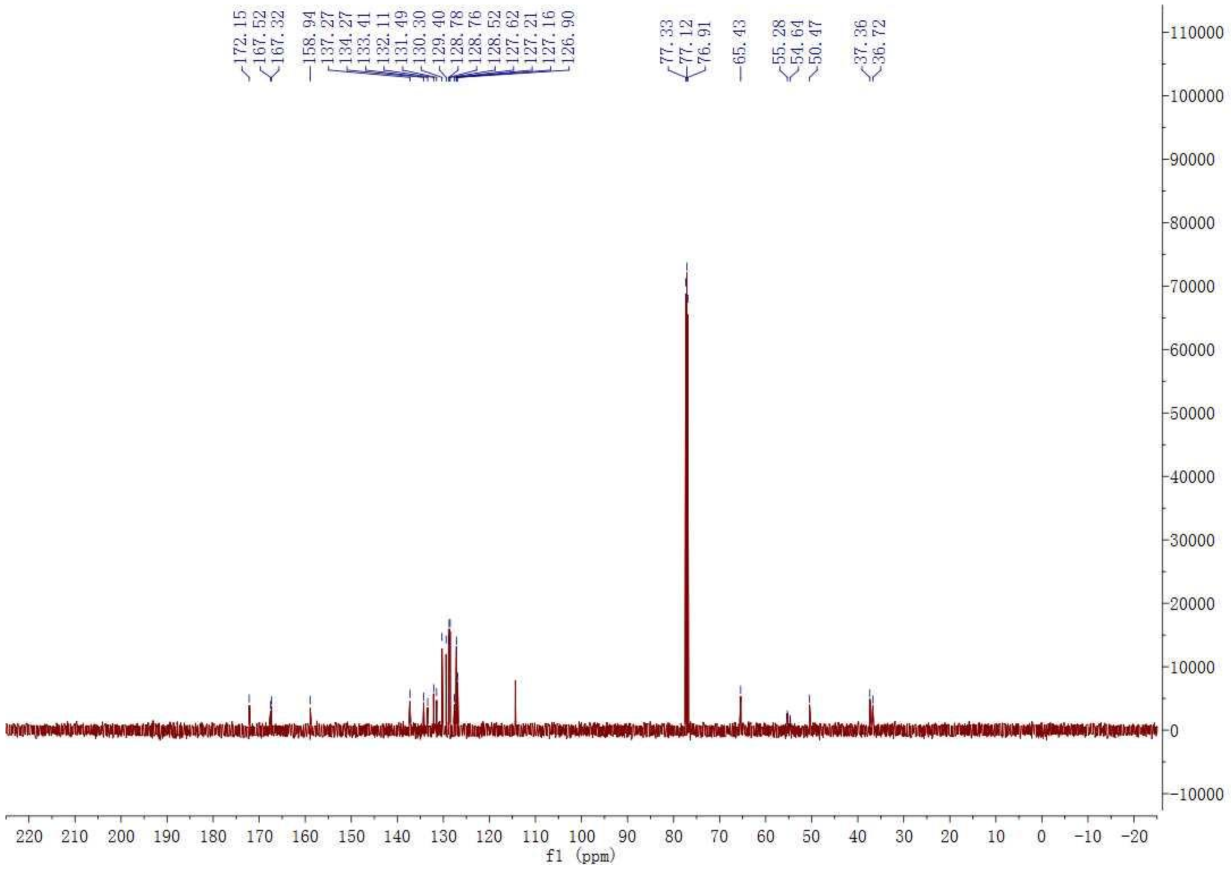

Figure S3. HMQC (DMSO-d_6) spectrum of compound **1**.

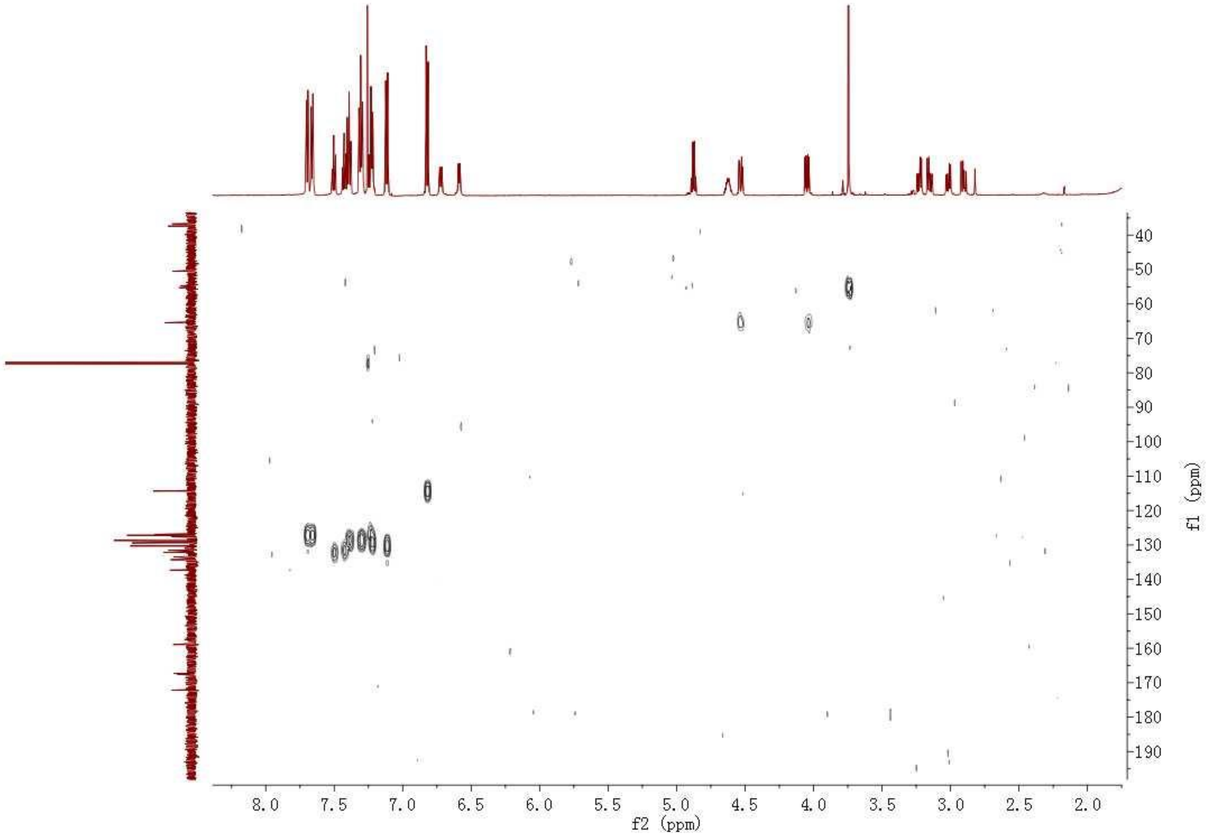

Figure S4. ^{1}H-^{1}H COSY (DMSO-d_6) spectrum of compound **1**.

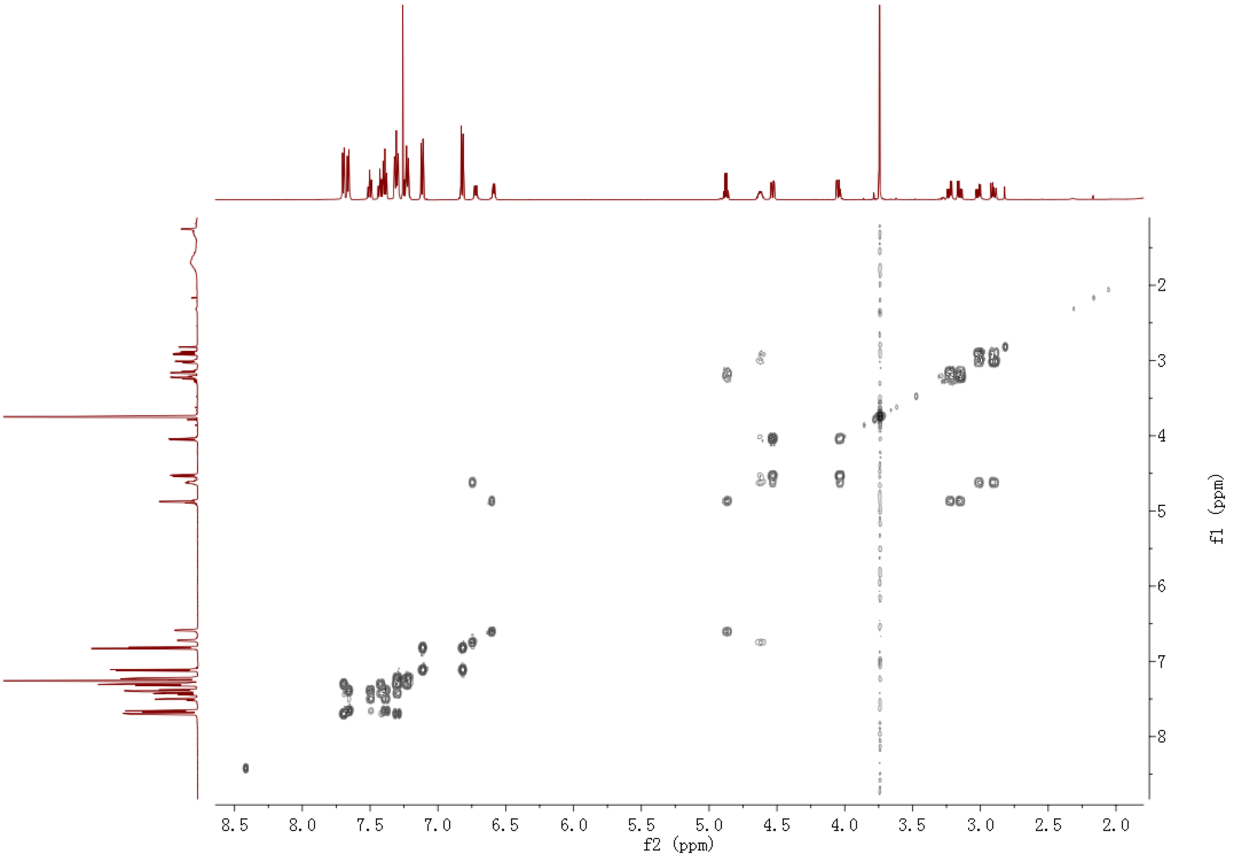

Figure S5. HMBC spectrum (DMSO-d_6) of compound **1**.

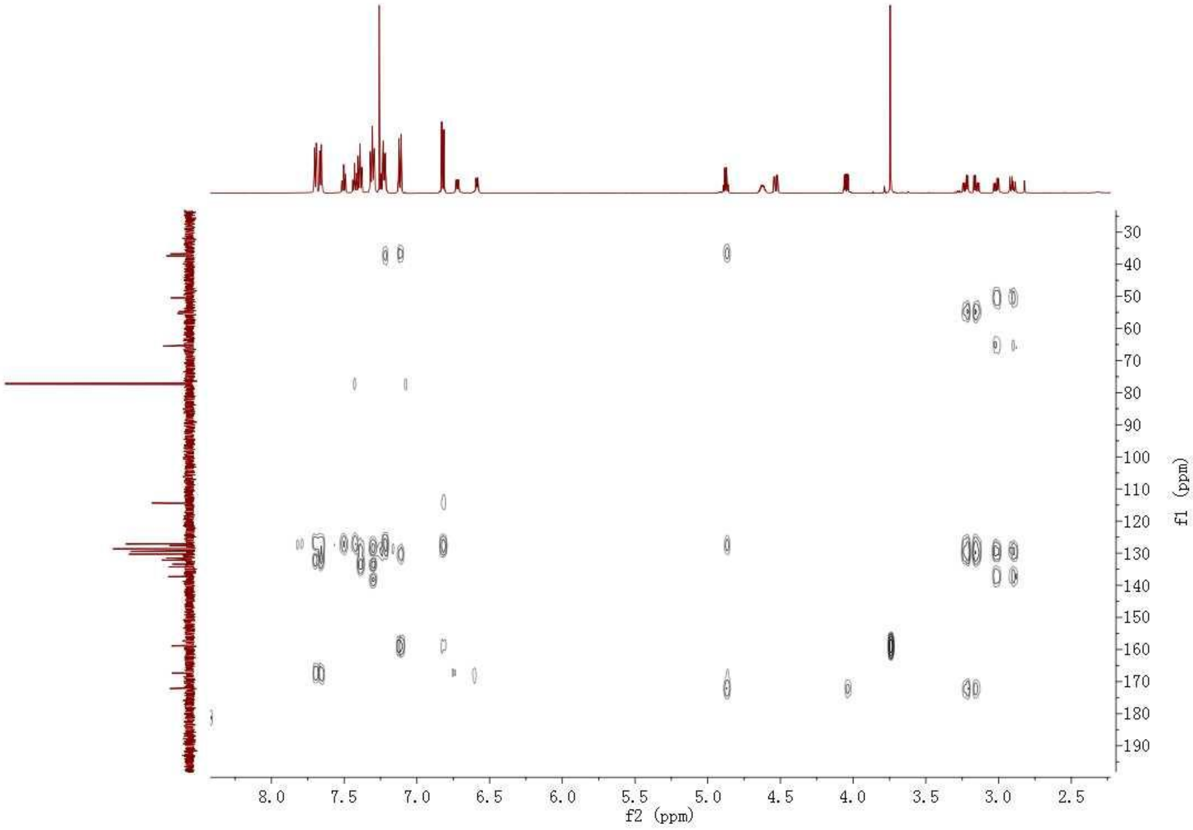

Figure S6. HRESIMS spectrum of compound **1**.

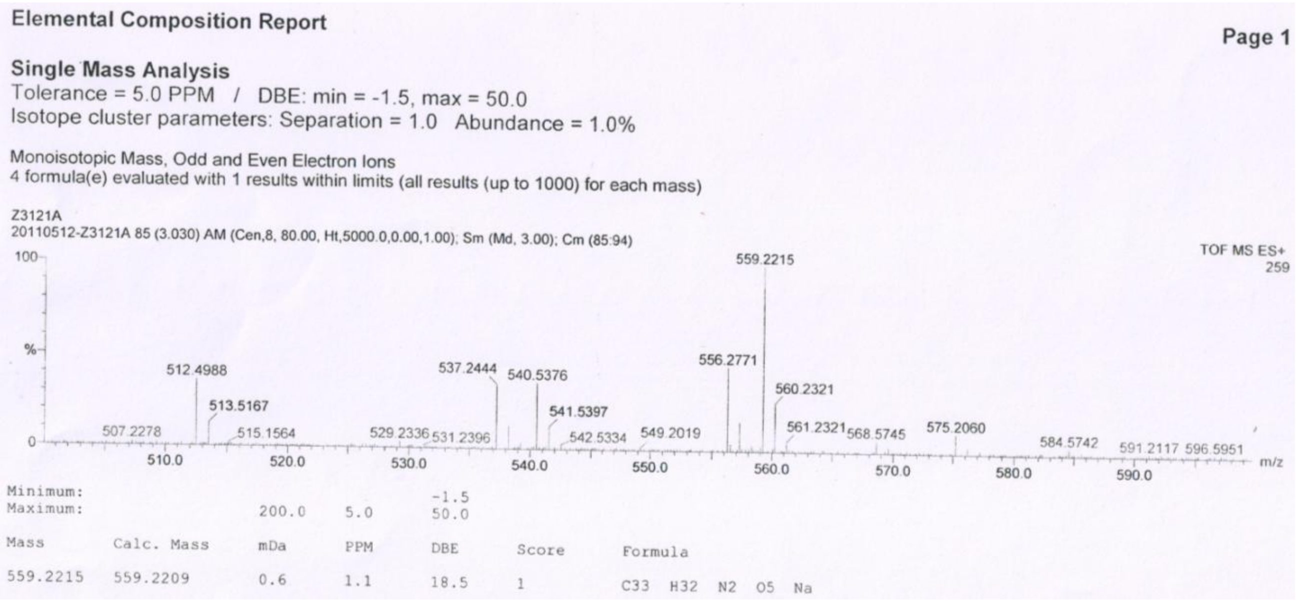

Figure S7. ^{1}H NMR (600 MHz, $CDCl_3$) spectrum of compound **3**.

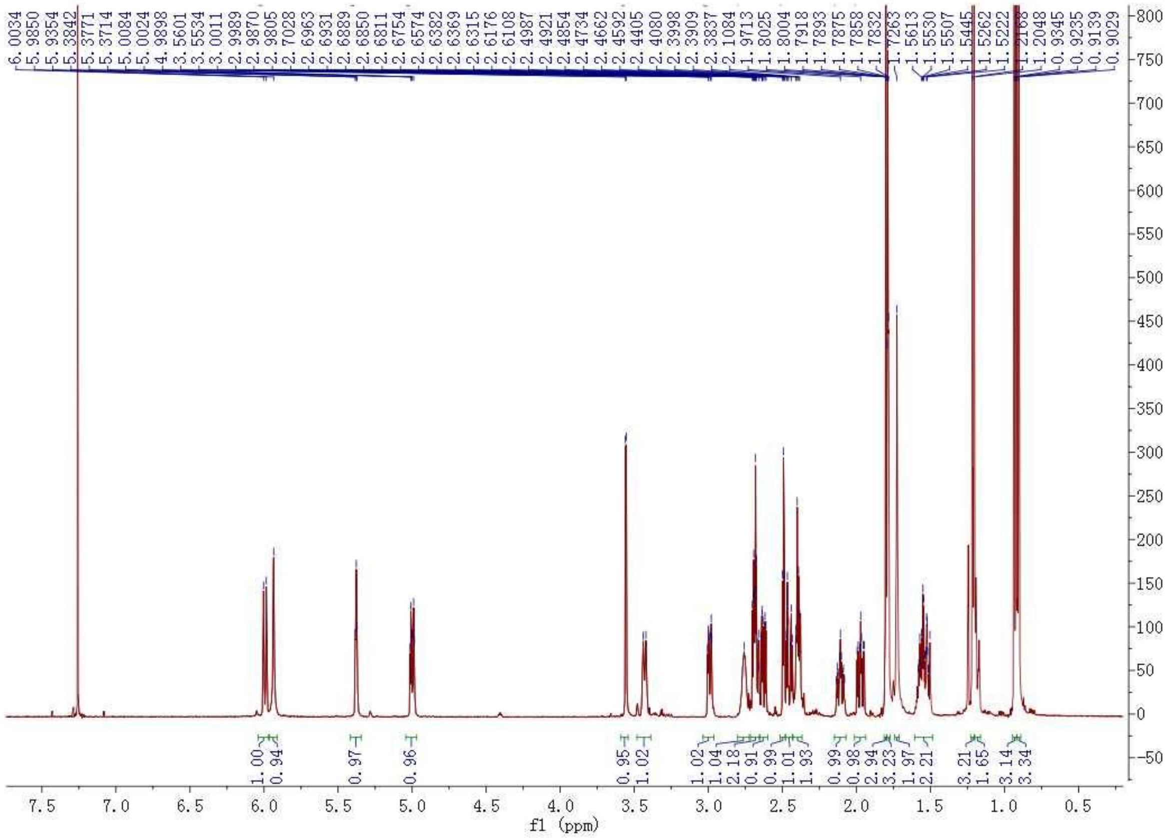

Figure S8. ^{13}C NMR (150 MHz, $CDCl_3$) spectrum of compound **3**.

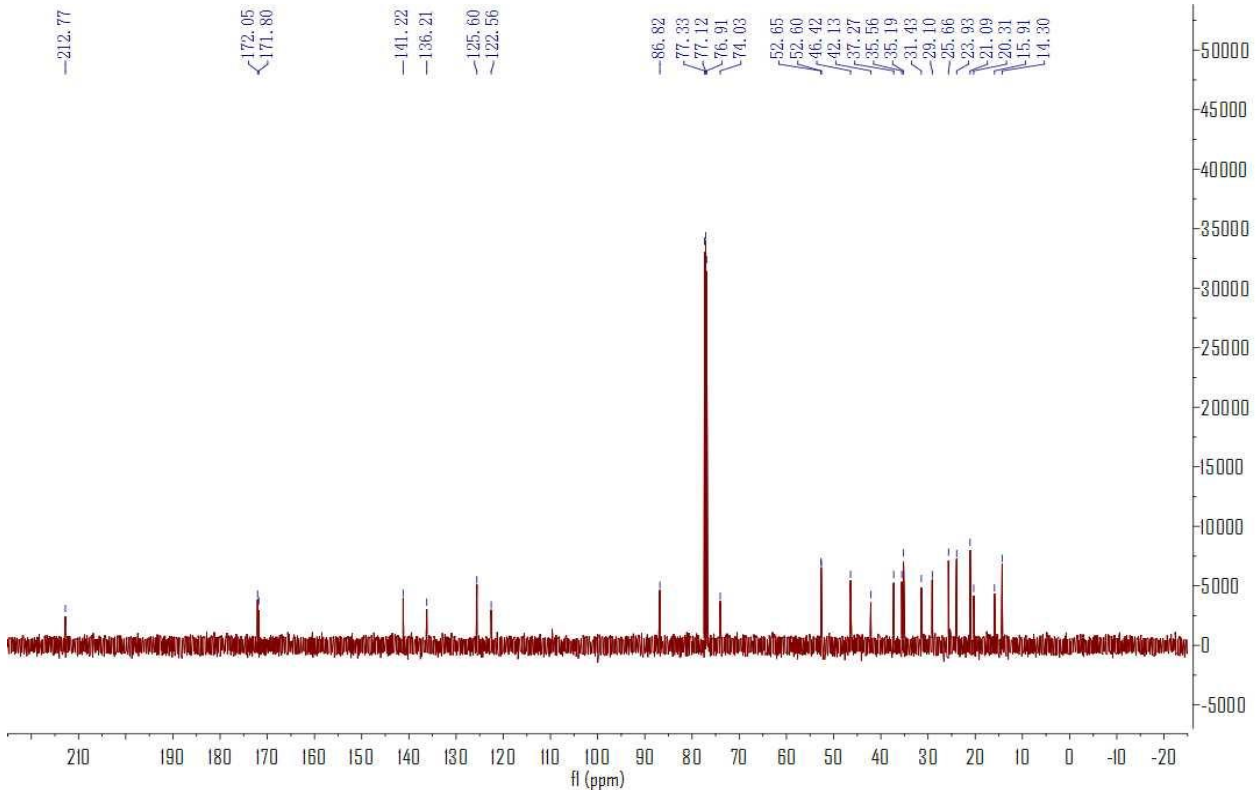

Figure S9. HMQC ($CDCl_3$) spectrum of compound **3**.

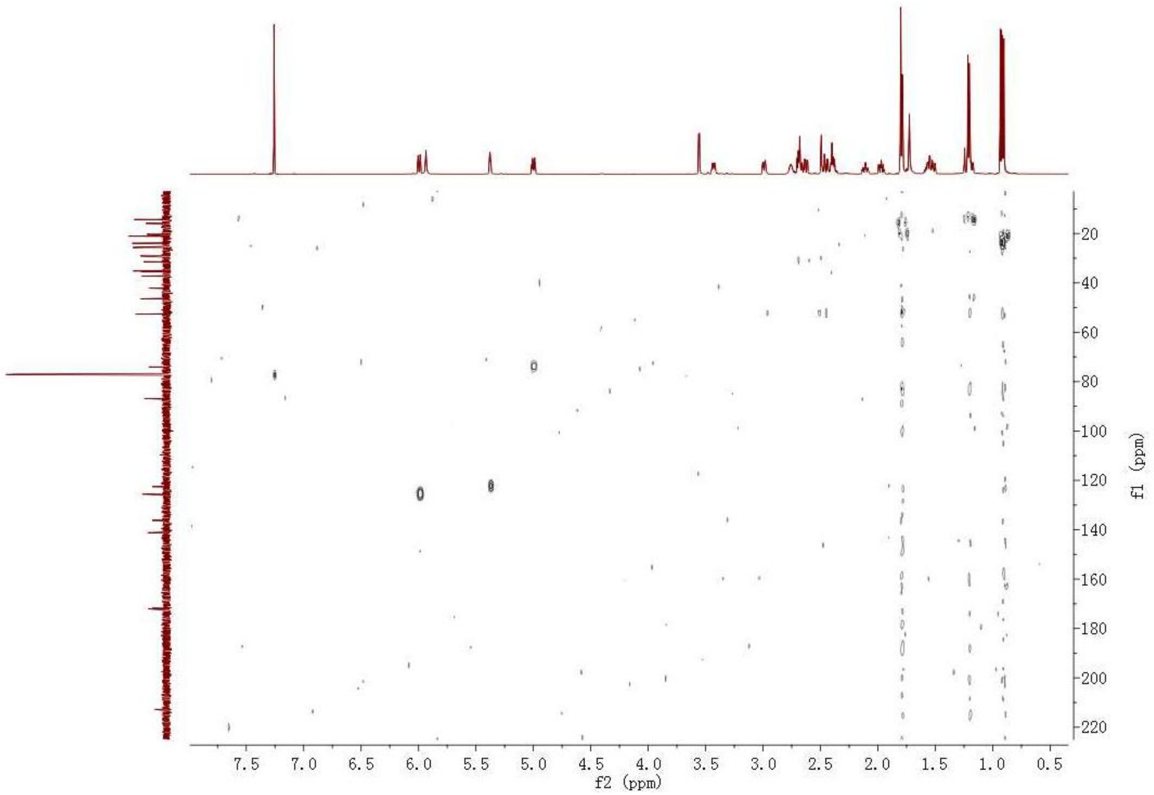

Figure S10. ^{1}H-^{1}H COSY ($CDCl_3$) spectrum of compound **3**.

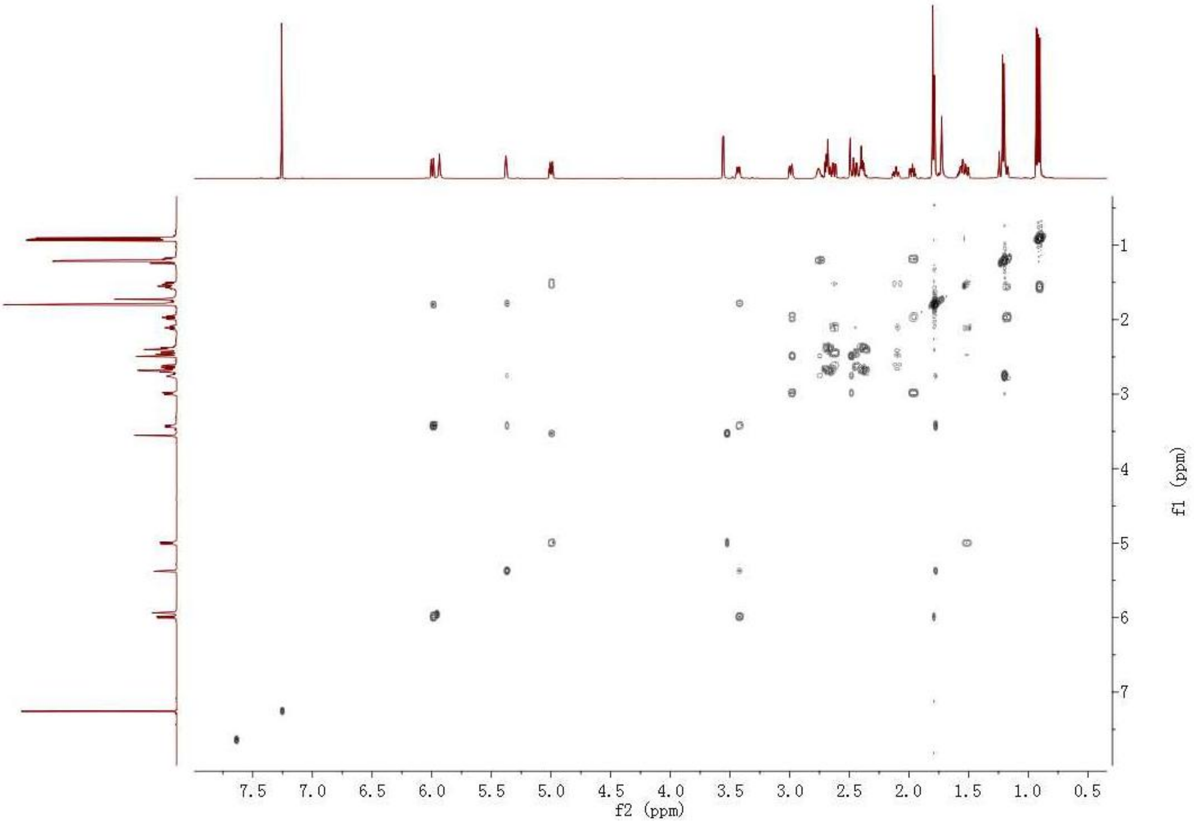

Figure S11. HMBC ($CDCl_3$) spectrum of compound **3**.

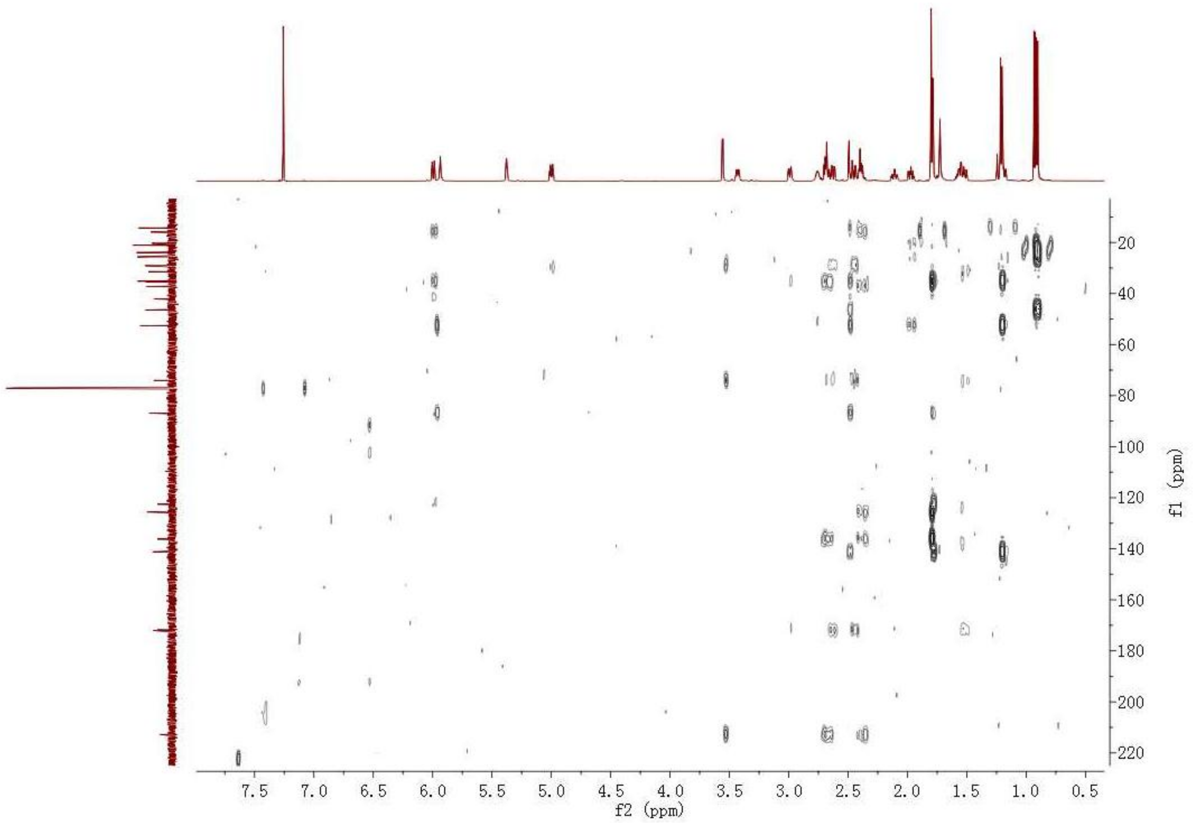

Figure S12. NOESY ($CDCl_3$) spectrum of compound **3**.

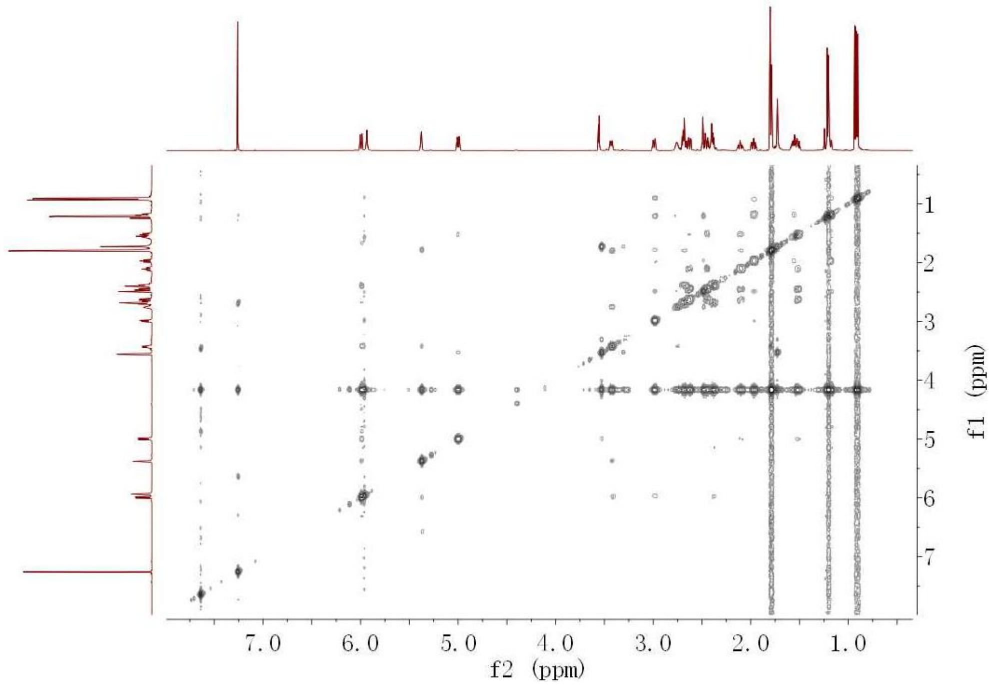

Figure S13. HRESIMS spectrum of compound **3**.

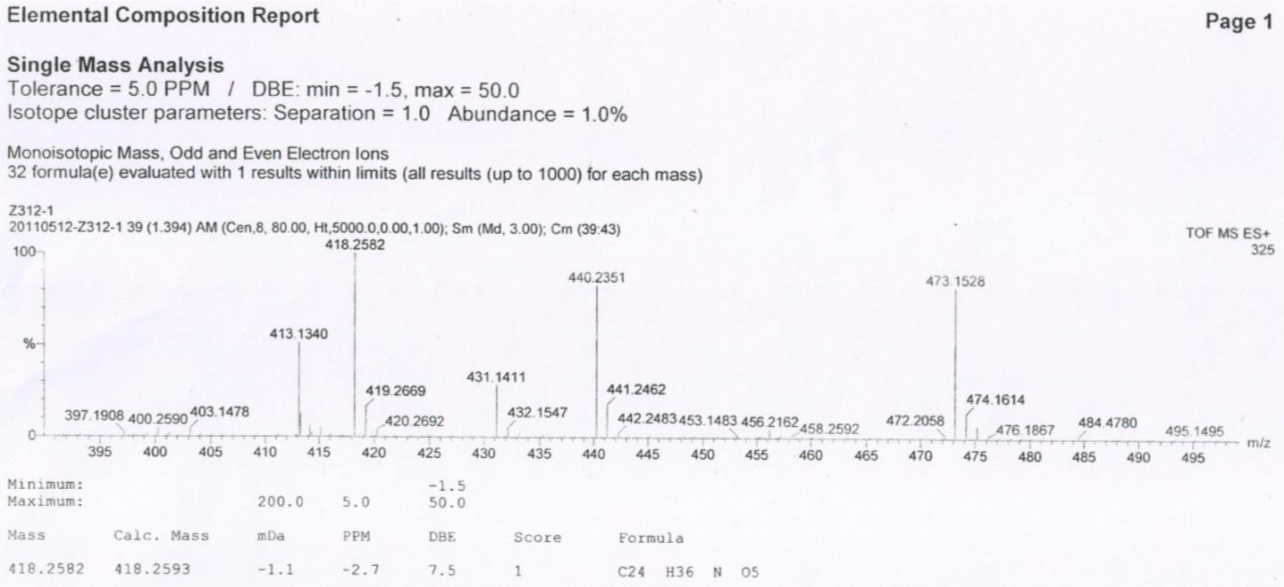

Figure S14. ^{1}H NMR (600 MHz, $CDCl_3$) spectrum of compound **4**.

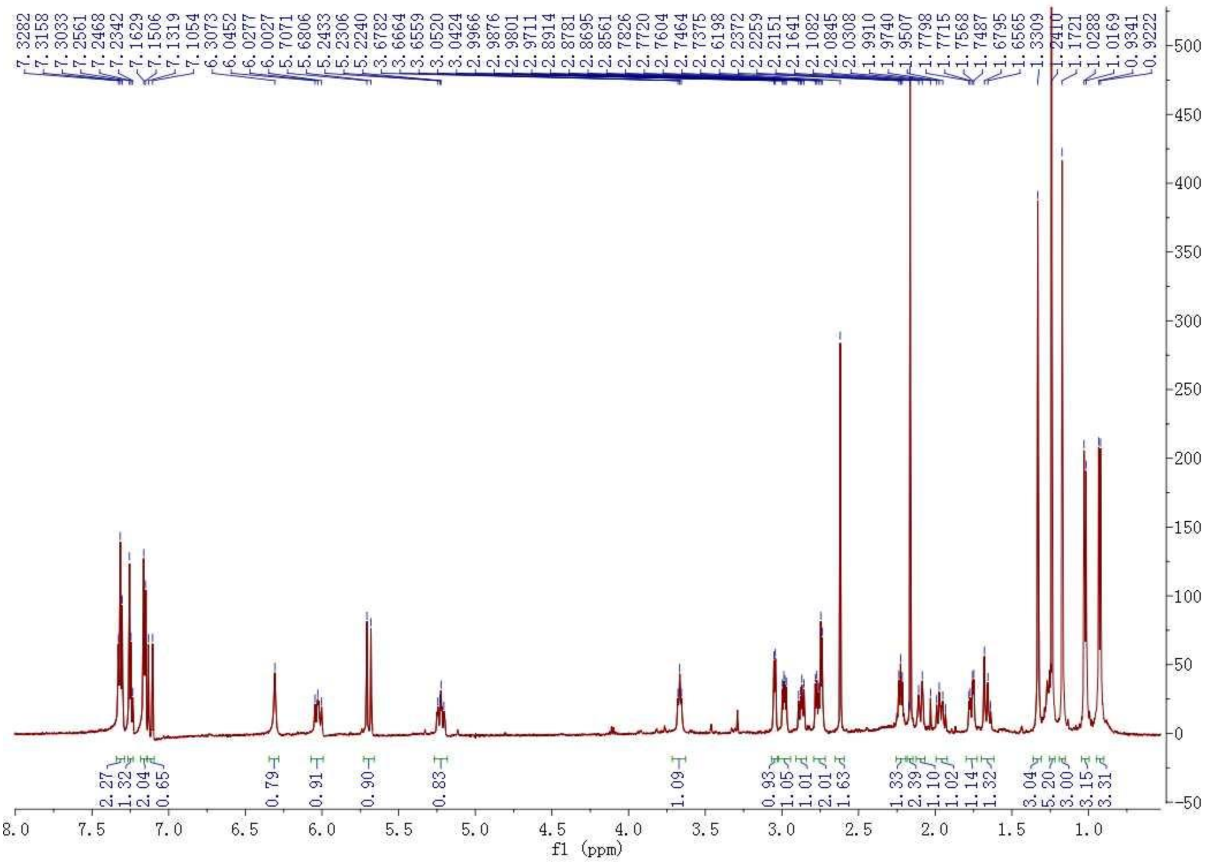

Figure S15. ^{13}C NMR (150 MHz, $CDCl_3$) spectrum of compound **4**.

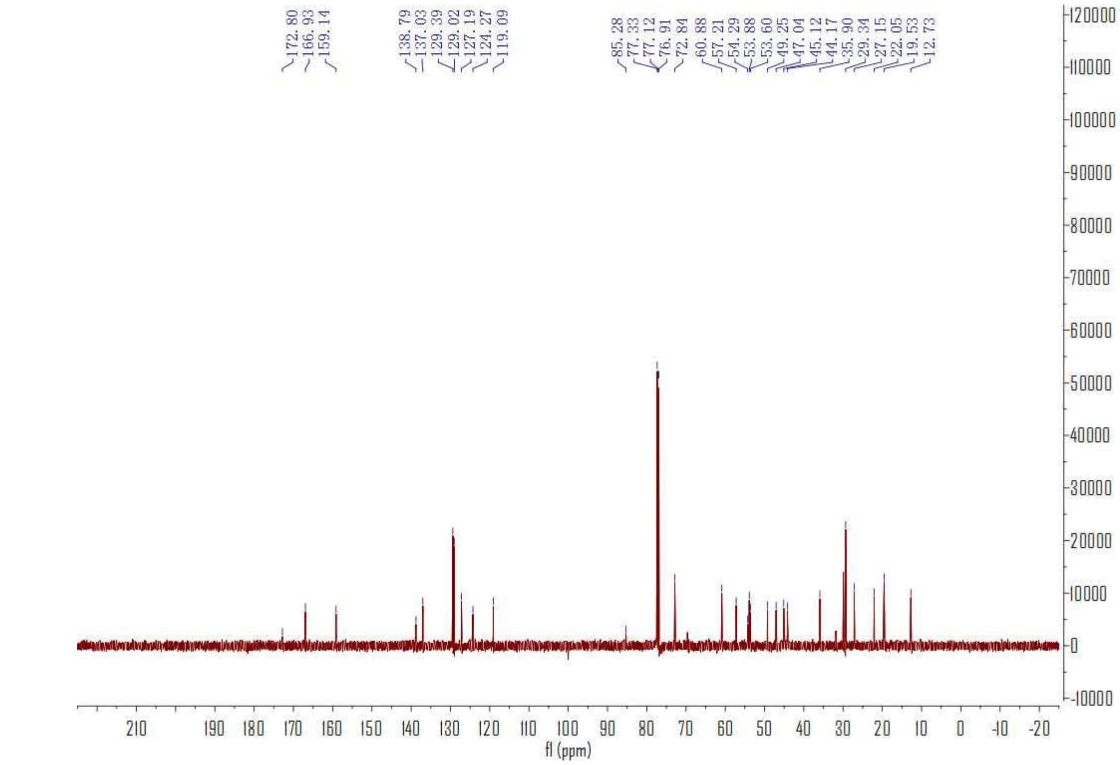

Figure S16. HMQC ($CDCl_3$) spectrum of compound **4**.

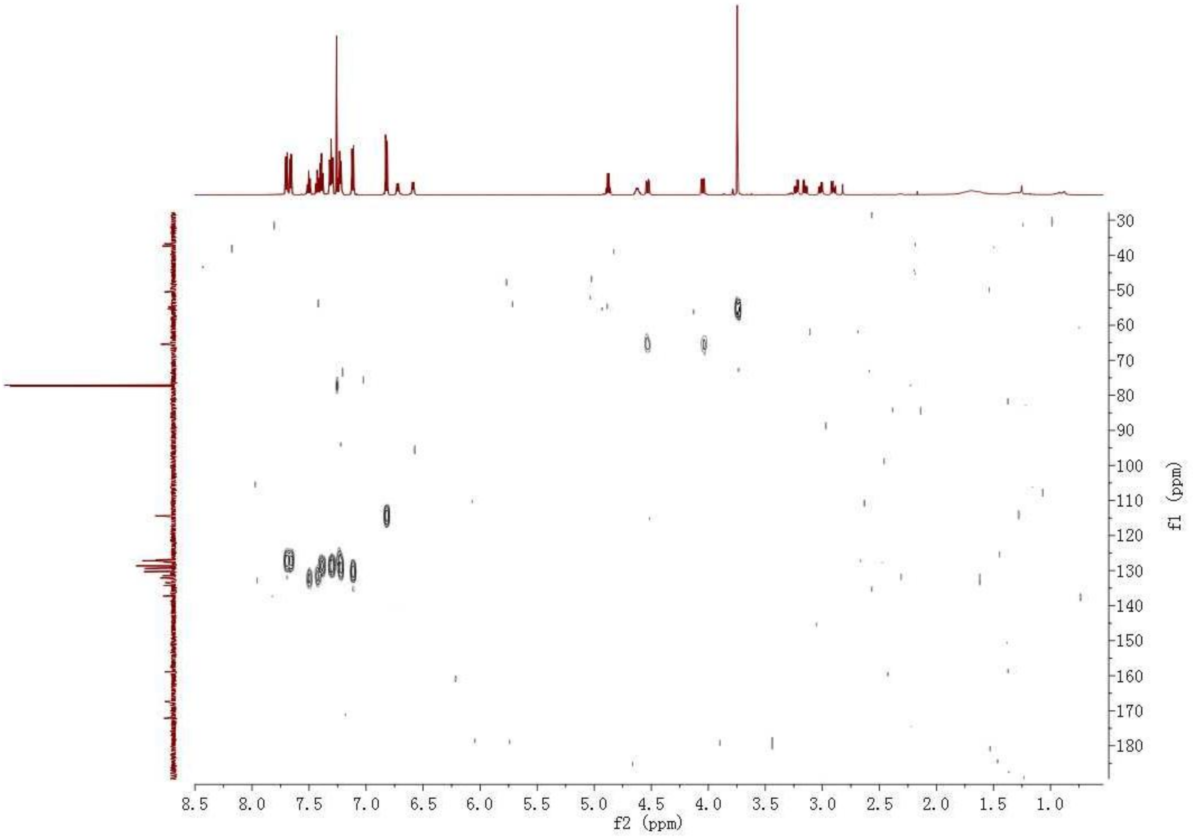

Figure S17. ^{1}H-^{1}H COSY ($CDCl_3$) spectrum of compound **4**.

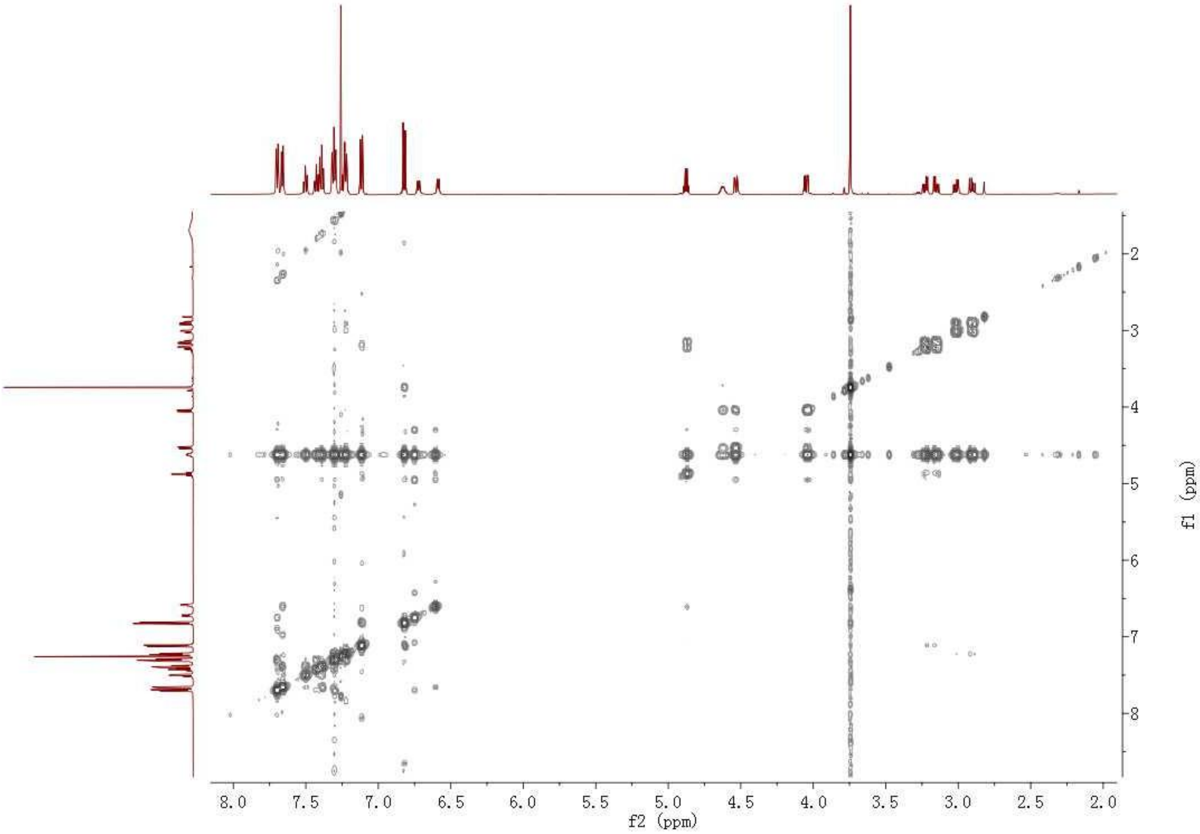

Figure S18. HMBC ($CDCl_3$) spectrum of compound **4**.

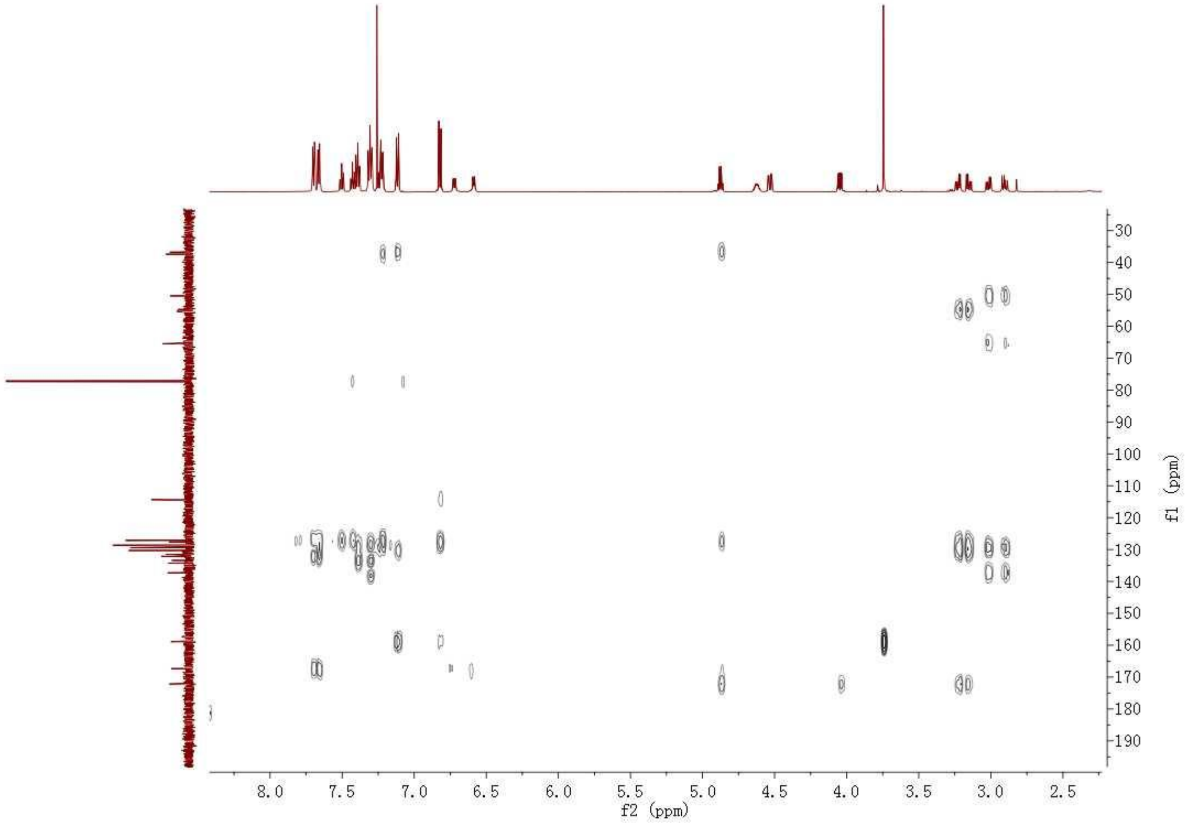

Figure S19. HRESIMS spectrum of compound **4**.

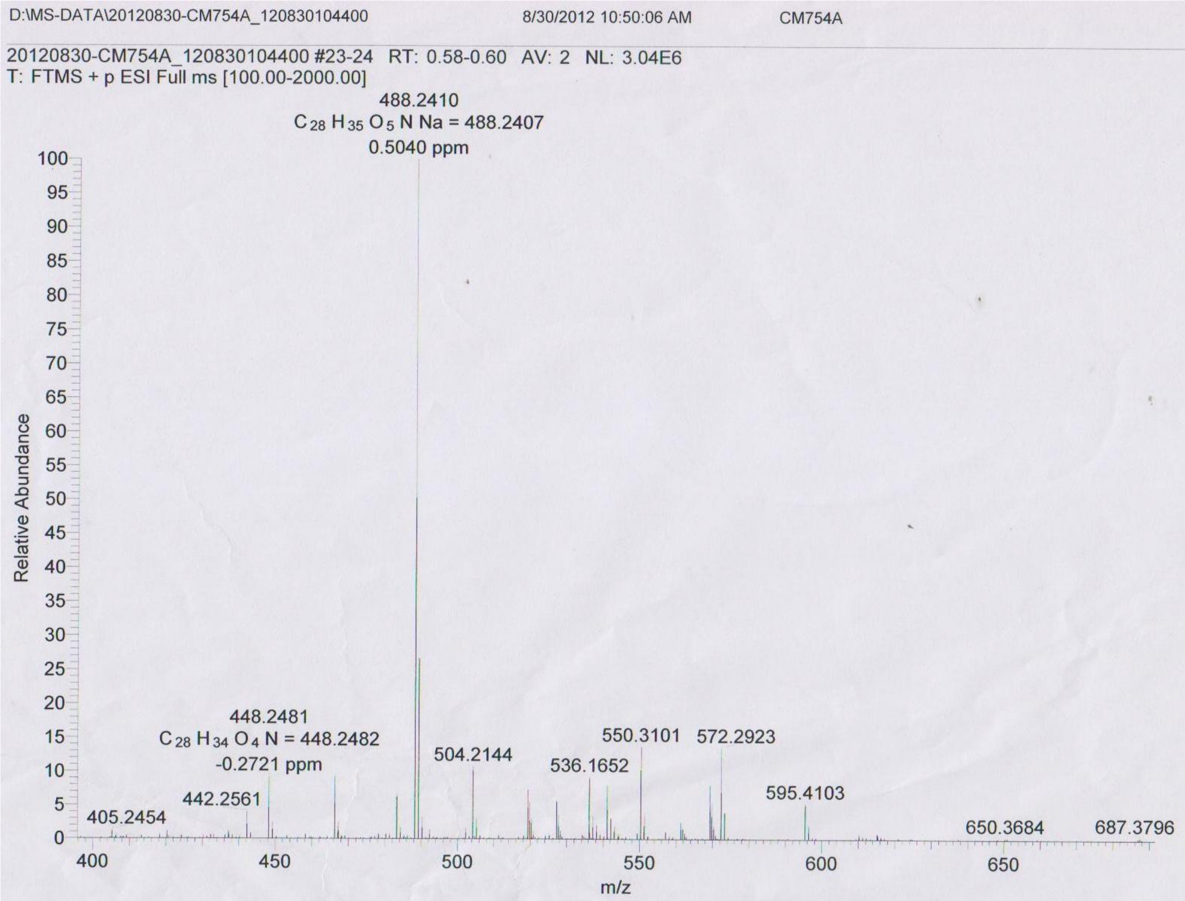

Figure S20. ^{1}H NMR (400 MHz, Acetone-d_6) spectrum of compounds **13a-1/13b-1**.

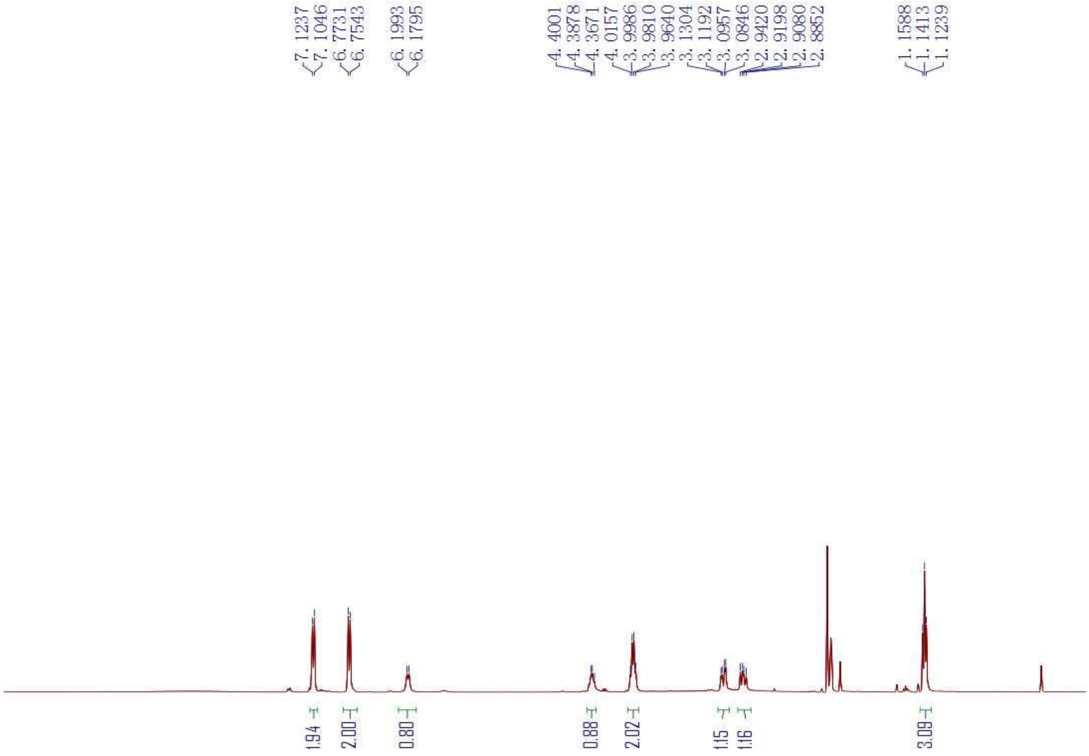

Figure S21. ^{13}C NMR (100 MHz, Acetone-d_6) spectrum of compounds **13a-1/13b-1**.

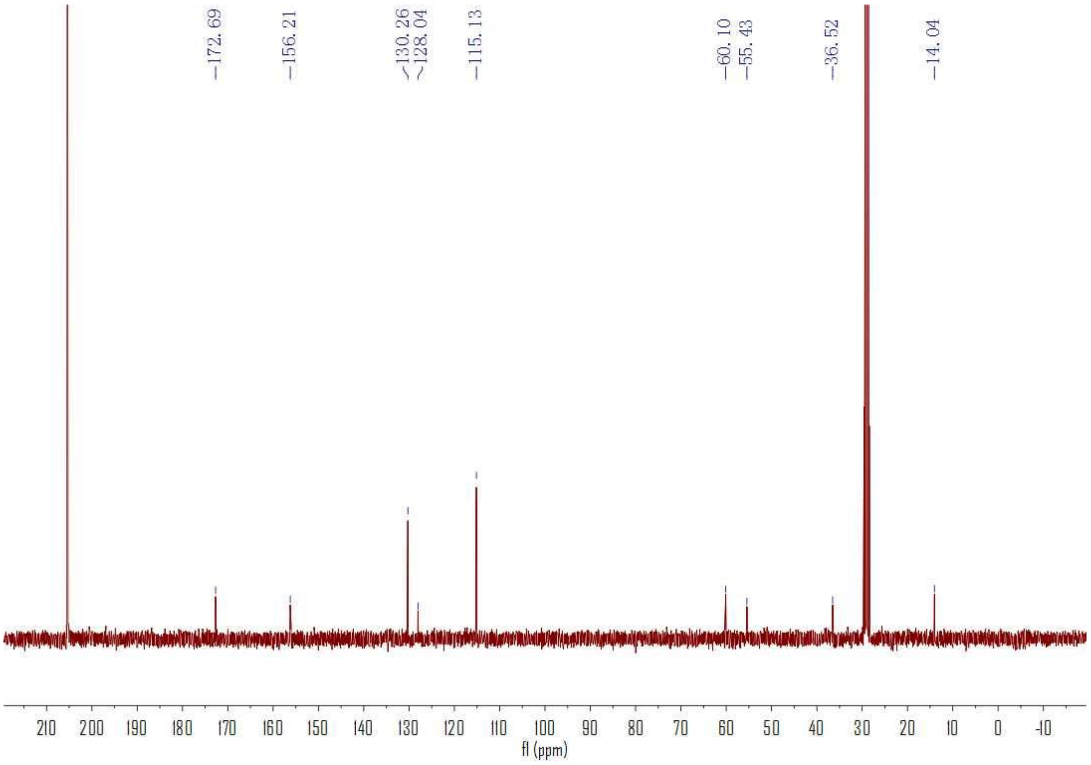

Figure S22. ESIMS spectrum of compounds **13a-1/13b-1**.

Mass Spectrum List Report

Analysis Info

Analysis Name	D:\Data\zheng caijuan\001.d	Acquisition Date	3/7/2013 3:02:25 PM
Method	DEF_MS.M	Operator	bruker
Sample Name	DL1	Instrument	HCT
Comment			

Acquisition Parameter

Ion Source Type	ESI	Ion Polarity	Negative	Alternating Ion Polarity	off
Mass Range Mode	Ultra Scan	Scan Begin	100 m/z	Scan End	500 m/z
Capillary Exit	-113.5 Volt	Skimmer	-40.0 Volt	Trap Drive	46.3
Accumulation Time	139 µs	Averages	3 Spectra	Auto MS/MS	off

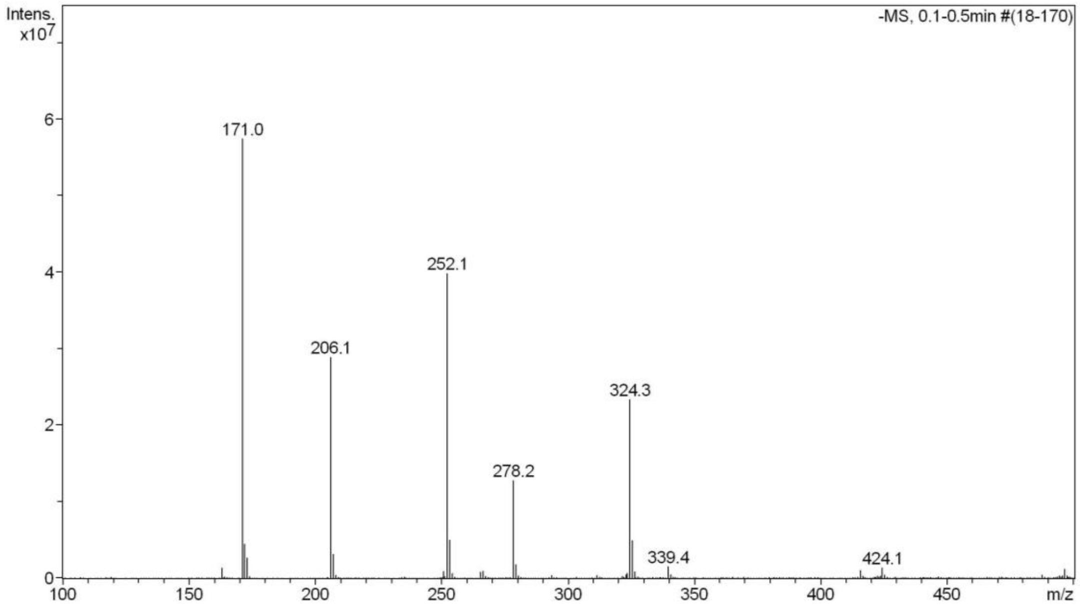

Figure S23. ^{1}H NMR (400 MHz, Acetone-d_6) spectrum of compounds **13a-2/13b-2**.

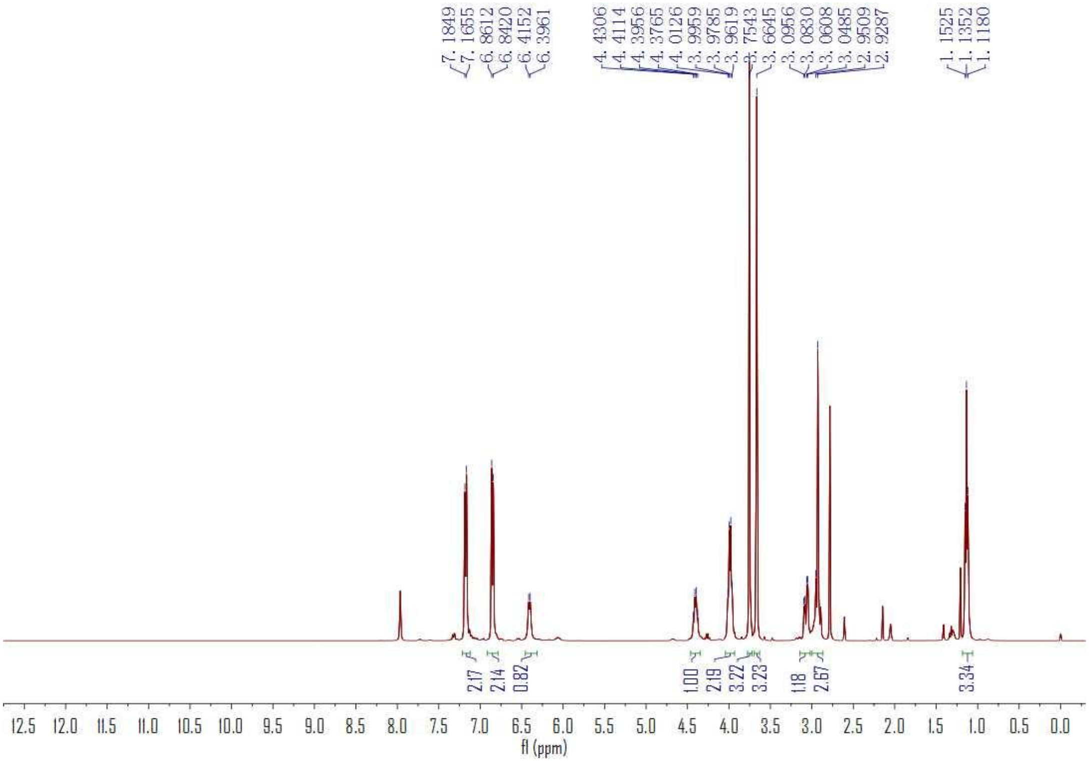

Figure S24. ^{13}C NMR (100 MHz, Acetone-d_6) spectrum of compounds **13a-2/13b-2**.

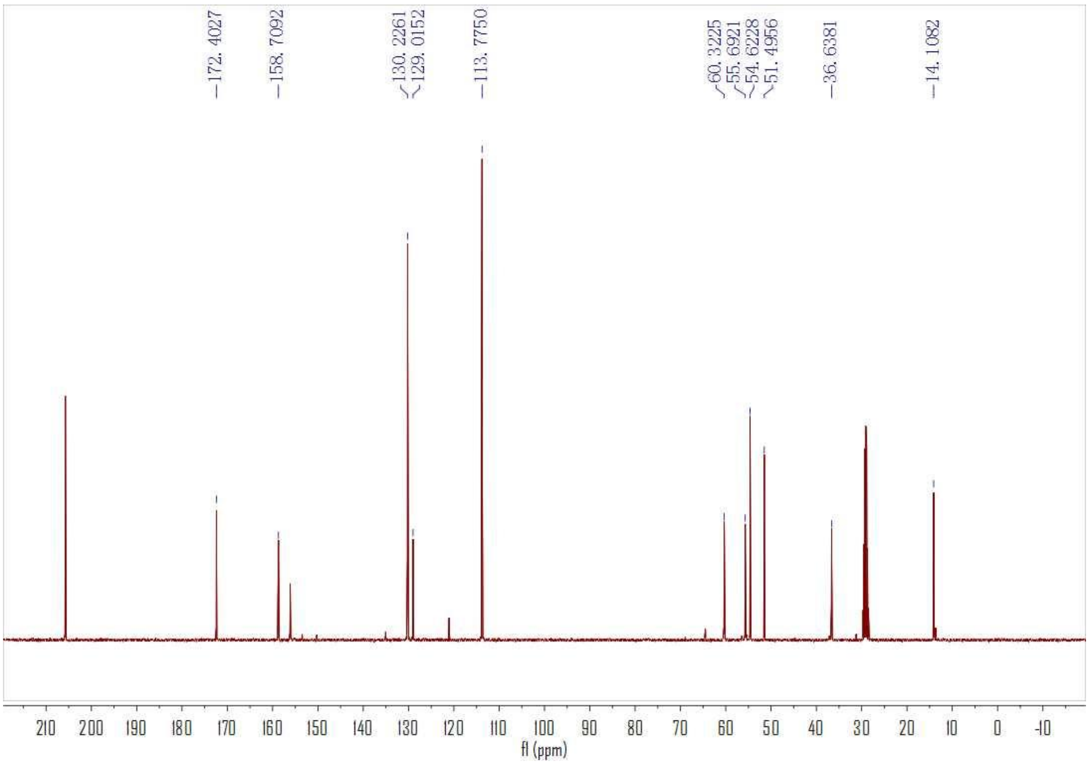

Figure S25. ESIMS spectrum of compounds **13a-2/13b-2**.

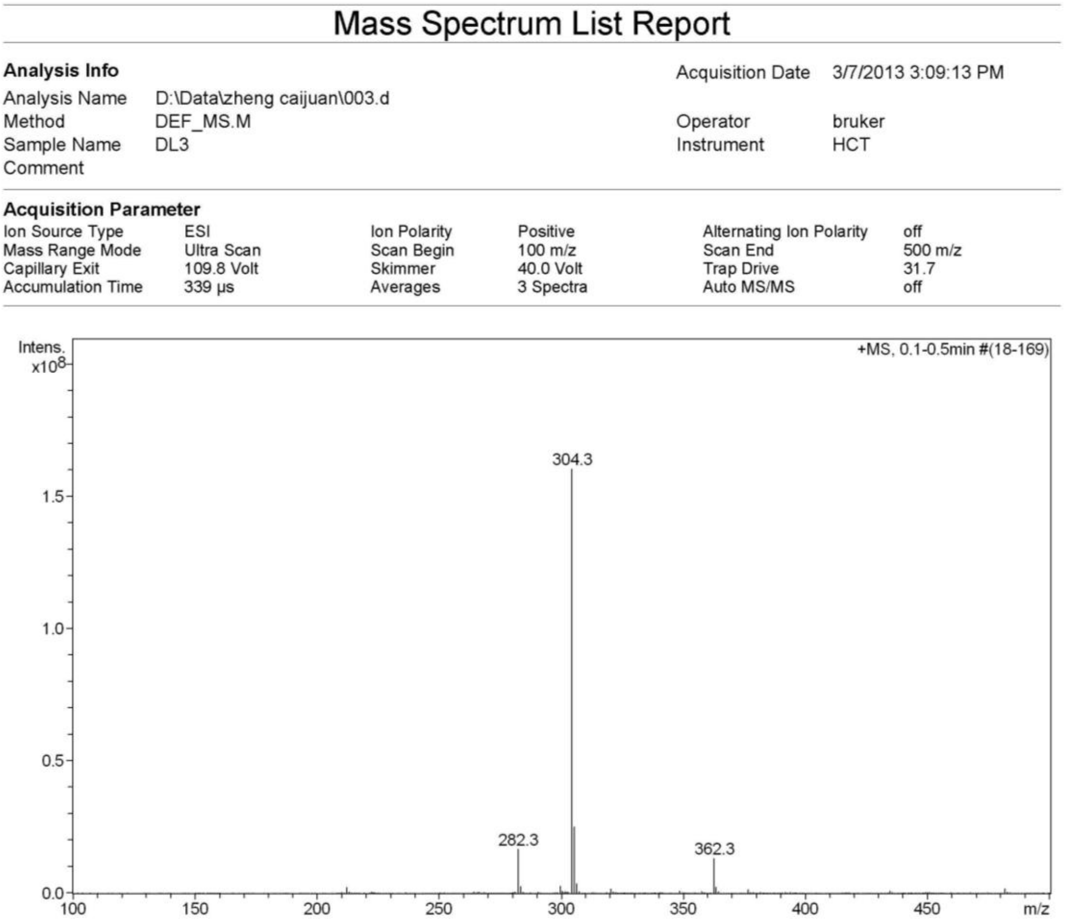

Figure S26. ^{1}H NMR (400 MHz, Acetone-d_6) spectrum of compounds **13a-3/13b-3**.

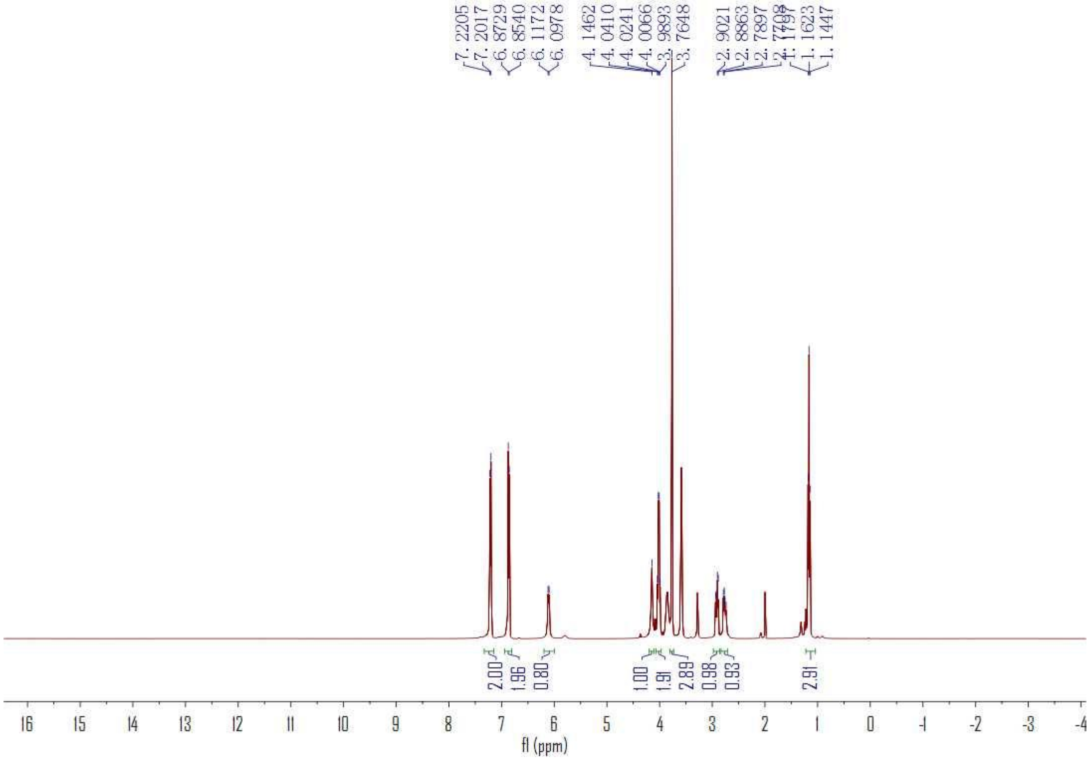

Figure S27. ^{13}C NMR (100 MHz, Acetone-d_6) spectrum of compounds **13a-3/13b-3**.

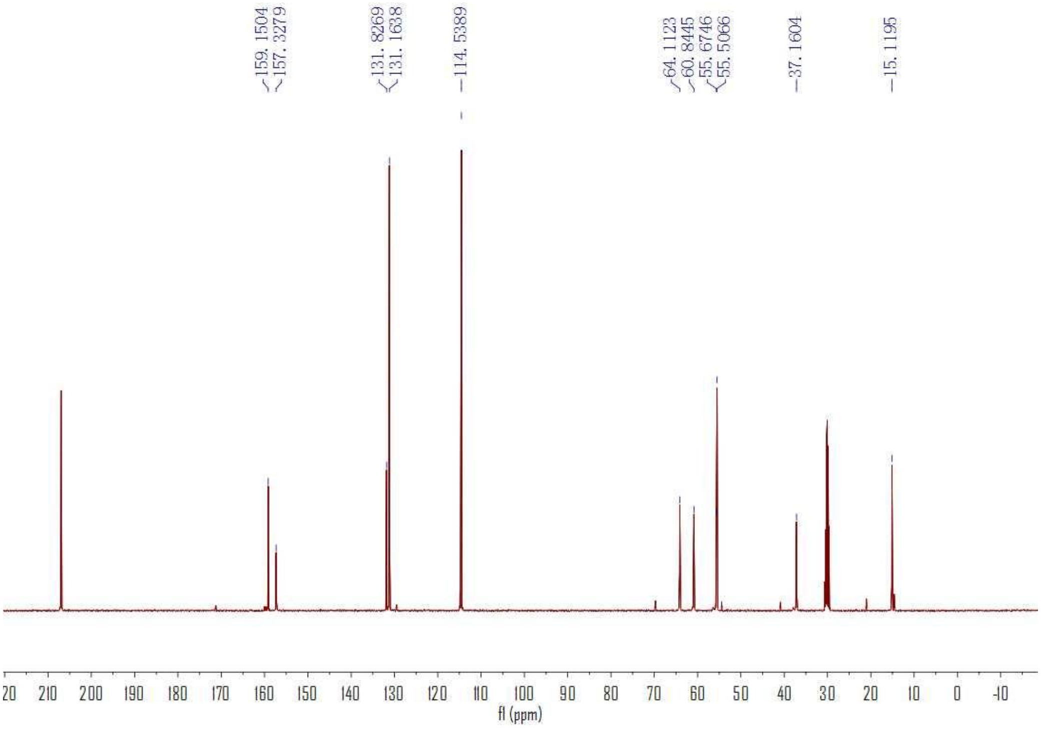

Figure S28. ESIMS spectrum of compounds **13a-3/13b-3**.

Mass Spectrum List Report

Analysis Info

Analysis Name	D:\Data\zheng caijuan\002.d	Acquisition Date	3/7/2013 3:06:39 PM
Method	DEF_MS.M	Operator	bruker
Sample Name	DL2	Instrument	HCT
Comment			

Acquisition Parameter

Ion Source Type	ESI	Ion Polarity	Negative	Alternating Ion Polarity	off
Mass Range Mode	Ultra Scan	Scan Begin	100 m/z	Scan End	500 m/z
Capillary Exit	-113.5 Volt	Skimmer	-40.0 Volt	Trap Drive	46.3
Accumulation Time	250 µs	Averages	3 Spectra	Auto MS/MS	off

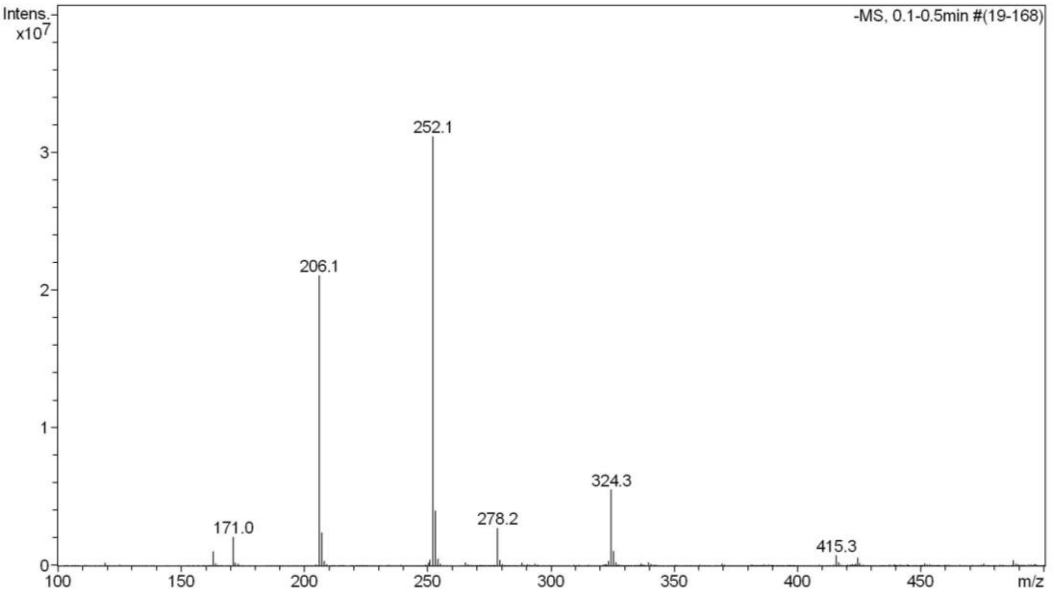

Figure S29. ^{1}H NMR (400 MHz, Acetone-d_6) spectrum of compounds **14a/14b**.

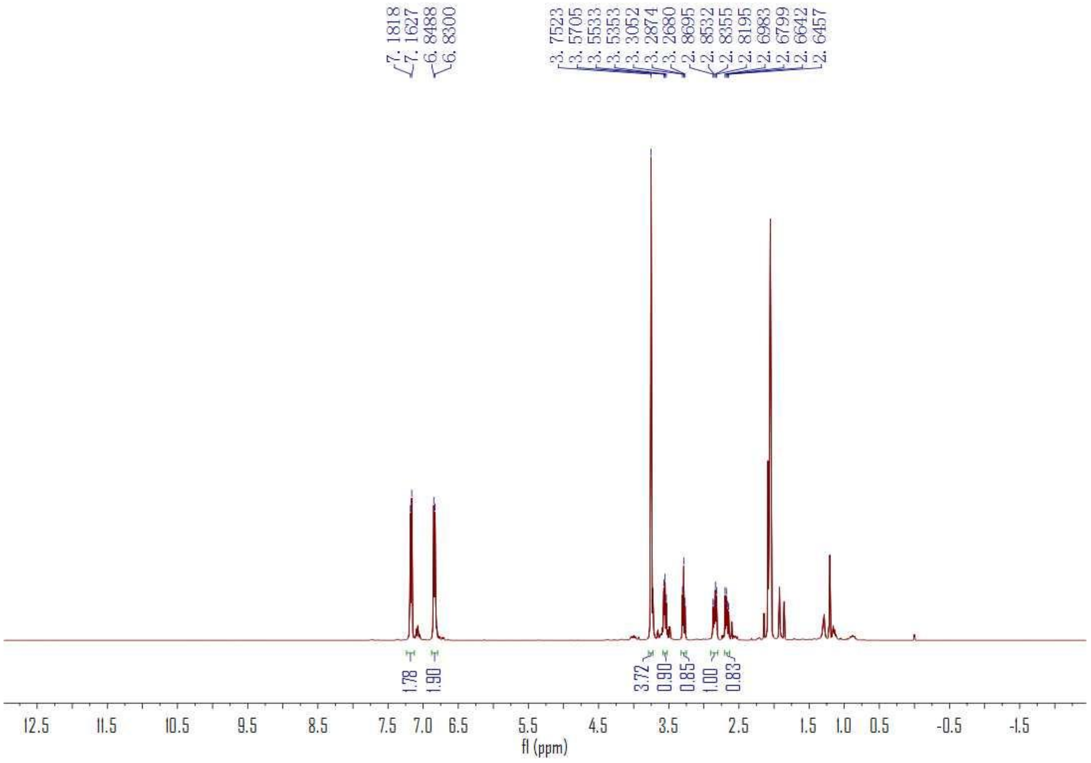

Figure S30. ^{13}C NMR (100 MHz, Acetone-d_6) spectrum of compounds **14a/14b**.

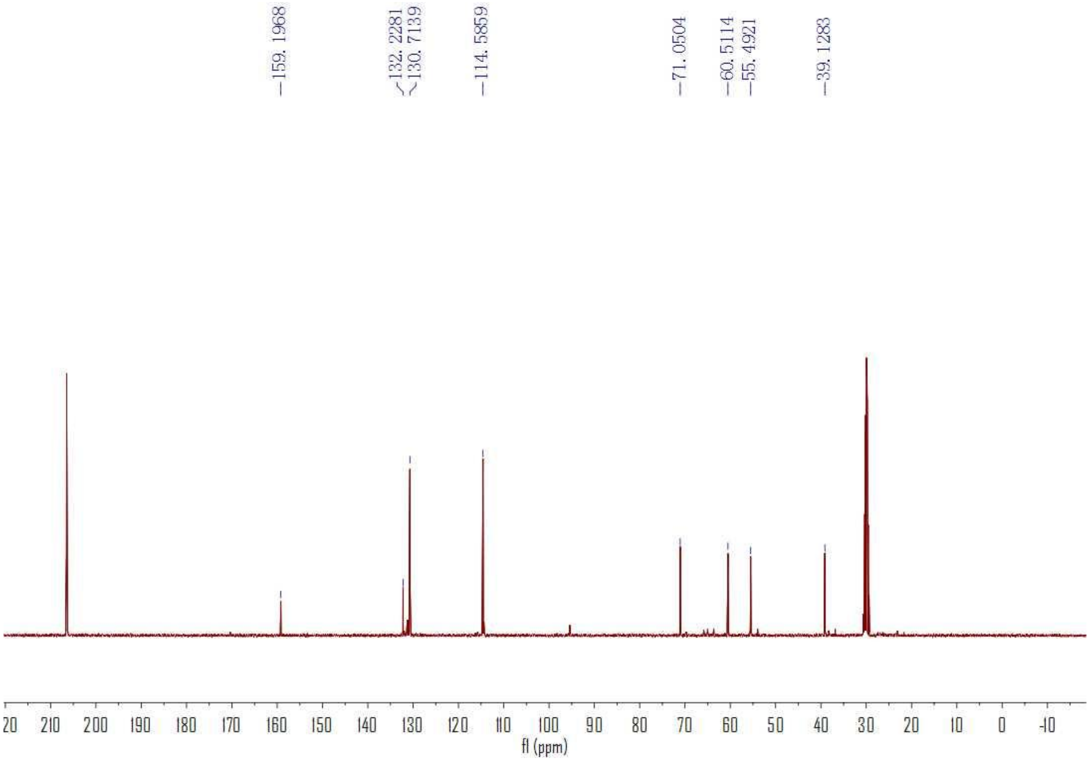

Figure S31. ESIMS spectrum of compounds **14a/14b**.

Mass Spectrum List Report

Analysis Info

Analysis Name	D:\Data\zheng caijuan\004.d	Acquisition Date	3/7/2013 3:11:22 PM
Method	DEF_MS.M	Operator	bruker
Sample Name	DL4	Instrument	HCT
Comment			

Acquisition Parameter

Ion Source Type	ESI	Ion Polarity	Positive	Alternating Ion Polarity	off
Mass Range Mode	Ultra Scan	Scan Begin	100 m/z	Scan End	500 m/z
Capillary Exit	109.8 Volt	Skimmer	40.0 Volt	Trap Drive	31.7
Accumulation Time	69 µs	Averages	3 Spectra	Auto MS/MS	off

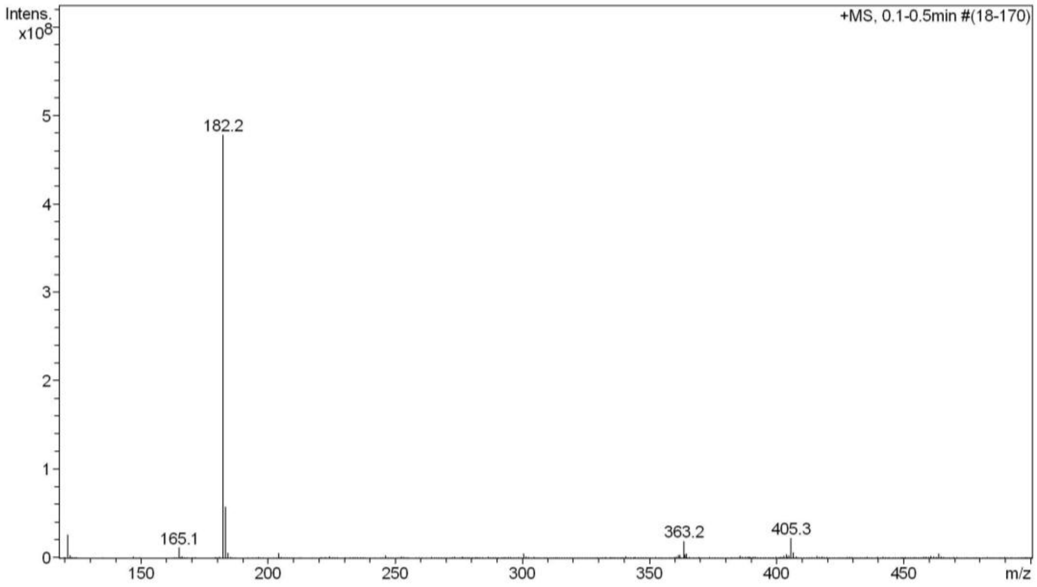

Figure S32. The determination of the absolute configuration of **1** by Marfey's Method (**A**–**G**) (HPLC analysis solvents: A, H_2O + 0.1% TFA, B, MeCN; linear gradient: 0 min, 25% B; 40 min, 60% B; 45 min, 100% B; temperature, 30 ℃; flow rate, 1 mL/min; UV detection at λ 340 nm; FDAA, 14.2 min).

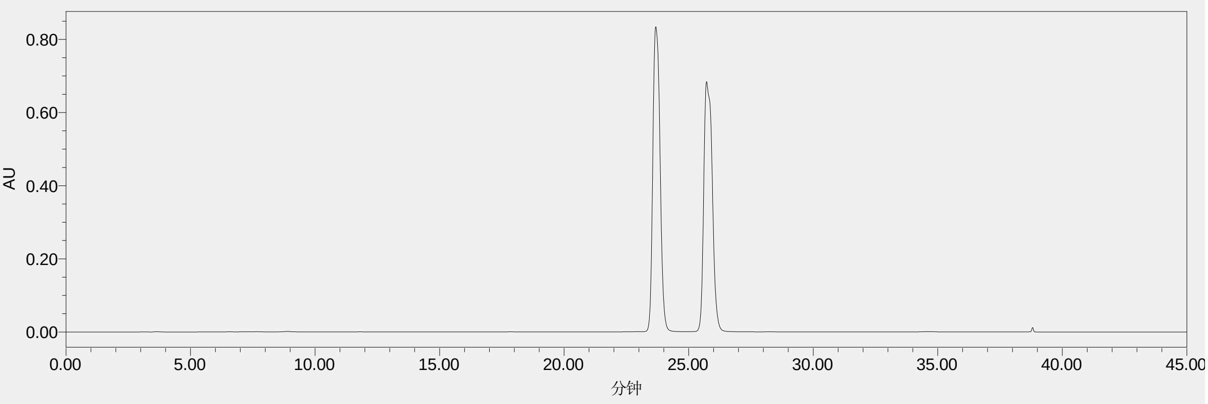

(**A**) FDAA derivatives of standard L- and D-Phe

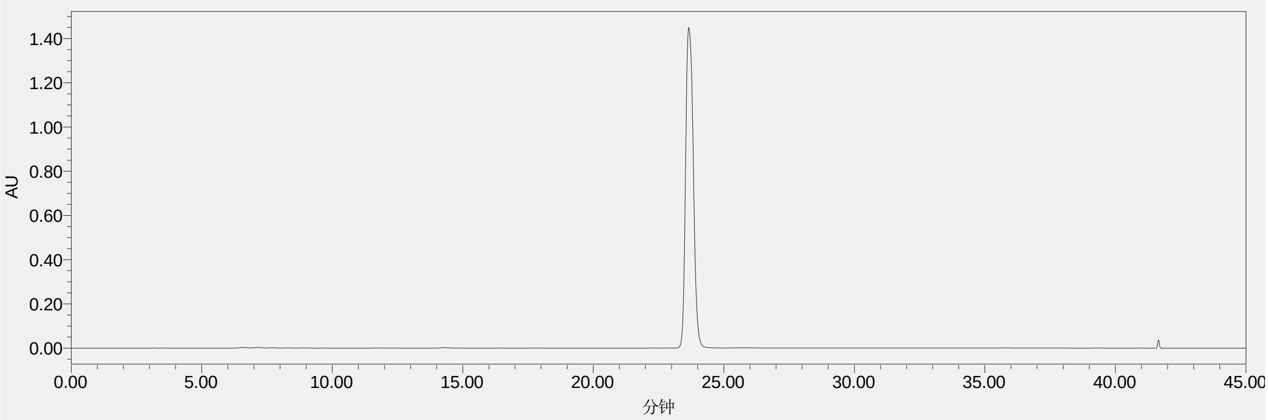

(**B**) FDAA derivative of standard L-Phe

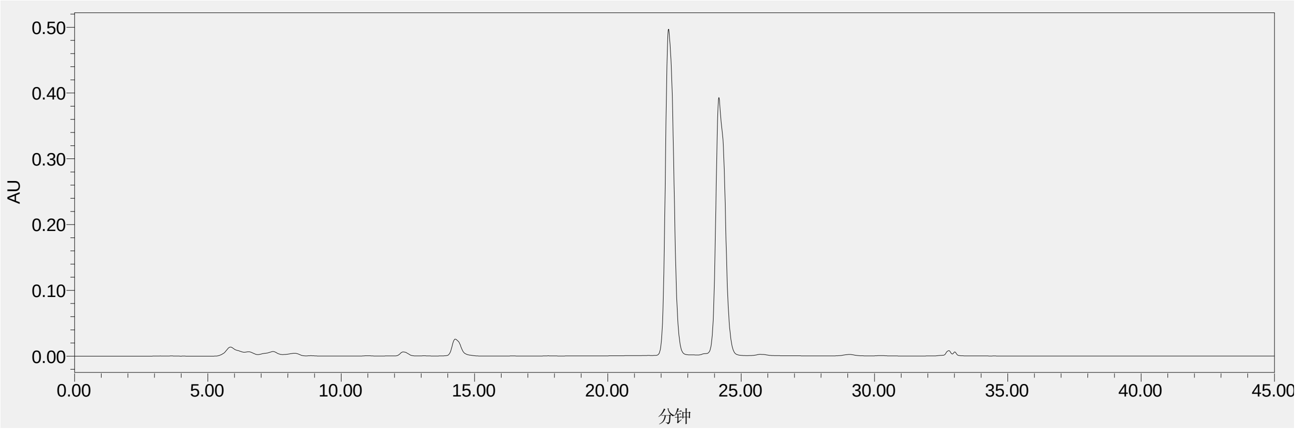

(**C**) FDAA derivatives of (*S*)-2-amino-3-(4-methoxyphenyl)-1-propanol and (*R*)-2-amino-3-(4-methoxyphenyl)-1-propanol (**14a**)

Figure S32. *Cont.*

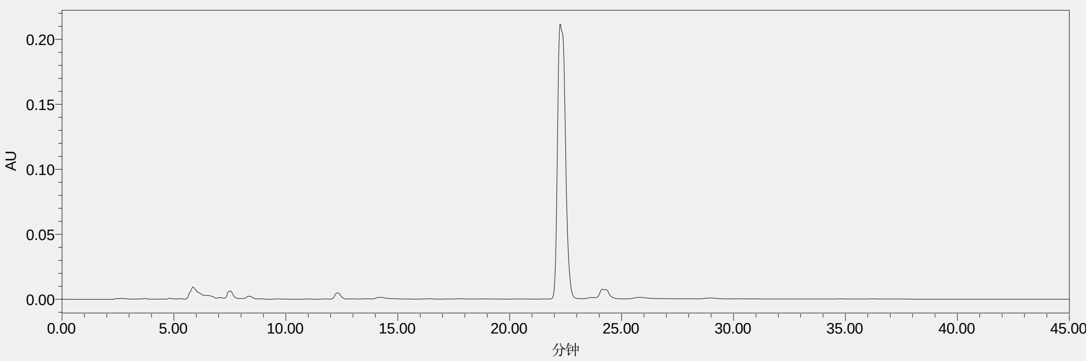

(**D**) FDAA derivative of (*S*)-2-amino-3-(4-methoxyphenyl)-1-propanol (**14b**)

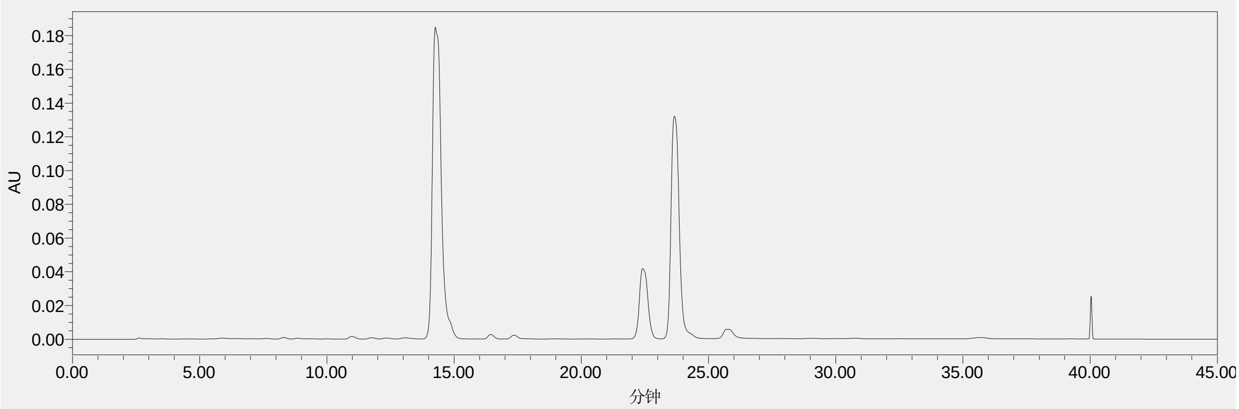

(**E**) FDAA derivatives of the hydrolysates from **1**

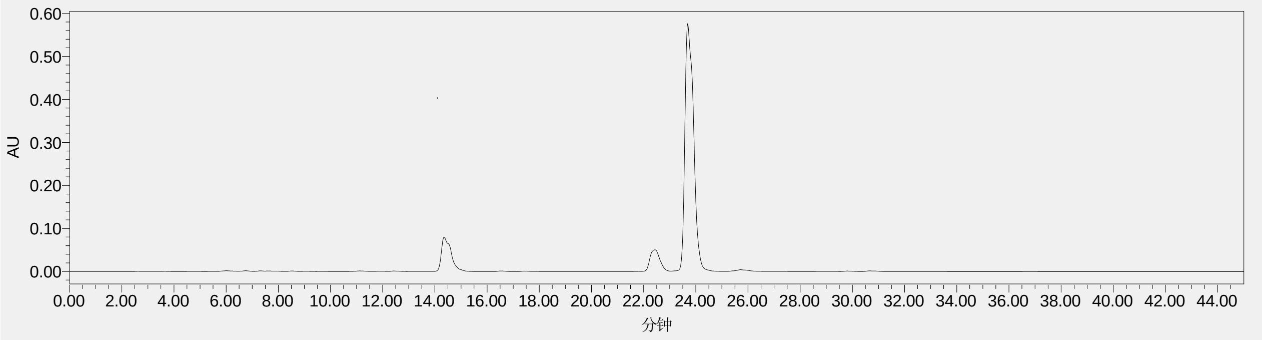

(**F**) Co-injection of FDAA derivatives of the hydrolysates from **1** with FDAA derivative of standard L-Phe

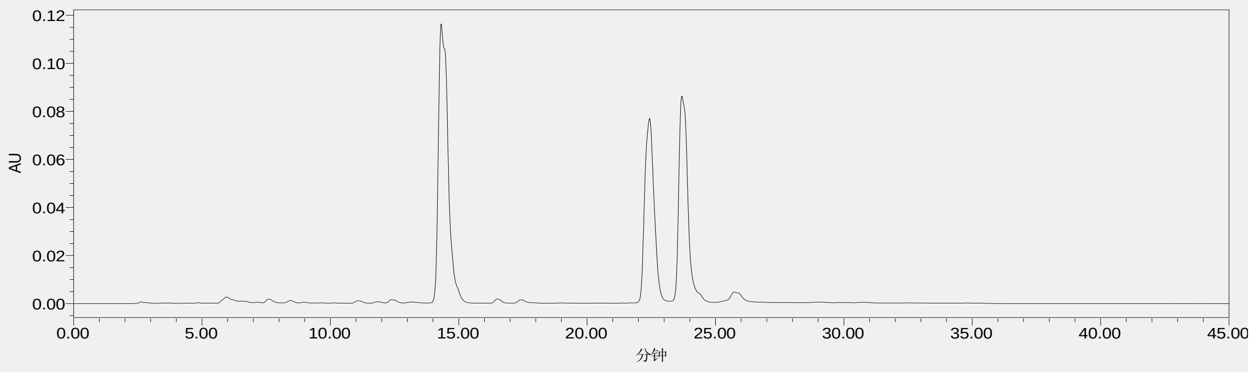

(**G**) Co-injection of FDAA derivatives of the hydrolysates from **1** with FDAA derivative of (*S*)-2-amino-3-(4-methoxyphenyl)-1-propanol (**14b**)

S1. The Spectroscopic Data of 13a-1, 13b-1, 13a-2, 13b-2, 13a-3, 13b-3, 14a and 14b

(*S*/*R*)-3-(4-Hydroxyphenyl)-2-[(ethoxycarbonyl)amino] propionic acid (13a-1) and (*S*)-3-(4-hydroxyphenyl)-2-[(ethoxycarbonyl)amino] propionic acid (13b-1): ^{1}H NMR (400 MHz, acetone-d_6, δ, ppm, *J*/Hz): 7.11 (2H, d, *J* = 7.6 Hz), 6.76 (2H, d, *J* = 7.6 Hz), 6.19 (1H, d, *J* = 8.0 Hz), 4.42 (1H, m), 3.99 (2H, q, *J* = 7.0 Hz), 3.11 (1H, dd, *J* = 13.9, 4.4 Hz), 2.91 (1H, dd, *J* = 13.9, 5.2 Hz), 1.14 (3H, t, *J* = 7.0 Hz). ^{13}C NMR (100 MHz, acetone-d_6, δ, ppm): 172.6 (C), 172.6 (C), 156.2 (C), 130.2 (CH), 130.2 (CH), 128.0 (C), 115.1 (CH), 115.1 (CH), 60.1 (CH_2), 55.4 (CH), 36.5 (CH_2), 14.0 (CH_3). ESIMS: 252.1 $[M - H]^-$.

Methyl (*S*/*R*)-2-[(ethoxycarbonyl)amino]-3-(4-methoxyphenyl) propanoate (13a-2) and methyl (*S*)-2-[(ethoxycarbonyl)amino]-3-(4-methoxyphenyl) propanoate (13b-2): ^{1}H NMR (400 MHz, acetone-d_6, δ, ppm, *J*/Hz): 7.18 (2H, d, *J* = 7.8 Hz), 6.85 (2H, d, *J* = 7.8 Hz), 6.41 (1H, d, *J* = 7.6 Hz), 4.40 (1H, dd, *J* = 14.0, 7.6 Hz), 3.99 (2H, q, *J* = 6.9 Hz), 3.75 (3H, s), 3.66 (3H, s), 3.07 (1H, dd, *J* = 13.9, 5.0 Hz), 2.94 (1H, dd, *J* = 13.9, 8.9 Hz), 1.14 (3H, t, *J* = 6.9 Hz). ^{13}C NMR (100 MHz, acetone-d_6, δ, ppm): 172.4 (C), 172.4 (C), 158.1 (C), 130.2 (CH), 130.2 (CH), 129.0 (C), 113.7 (CH), 113.7 (CH), 60.3 (CH_2), 55.6 (CH), 54.6 (CH_3), 51.5 (CH_3), 36.6 (CH_2), 14.1 (CH_3). ESIMS: 282.3 $[M + H]^+$.

(*S*/*R*)-2-[(Ethoxycarbonyl)amino]-3-(4-methoxyphenyl) propan-1-ol (13a-3) and (*S*)-2-[(ethoxycarbonyl)amino]-3-(4-methoxyphenyl) propan-1-ol (13b-3): ^{1}H NMR (400 MHz, acetone-d_6, δ, ppm, *J*/Hz): 7.21 (2H, d, *J* = 7.6 Hz), 6.86 (2H, d, *J* = 7.6 Hz), 6.11 (1H, d, *J* = 7.8 Hz), 4.15 (1H, m), 4.02 (2H, q, *J* = 7.0 Hz), 3.76 (3H, s), 3.38 (2H, m), 2.91 (1H, dd, *J* = 13.6, 6.4 Hz), 2.76 (1H, dd, *J* = 13.6, 7.8 Hz), 1.16 (3H, t, *J* = 7.0 Hz). ^{13}C NMR (100 MHz, acetone-d_6, δ, ppm): 159.1 (C), 157.3 (C), 131.8 (C), 131.1 (CH), 131.1 (CH), 114.5 (CH), 114.5 (CH), 64.1 (CH_2), 60.8 (CH_2), 55.6 (CH), 55.5 (CH_3), 37.1 (CH_2), 15.1 (CH_3). ESIMS: 252.1 $[M - H]^-$.

(*S*/*R*)-2-Amino-3-(4-methoxyphenyl)-1-propanol (14a) and (*S*)-2-amino-3-(4-methoxyphenyl)-1-propanol (14b): ^{1}H NMR (400 MHz, acetone-d_6, δ, ppm, *J*/Hz): 7.17 (2H, d, *J* = 7.5 Hz), 6.84 (2H, d, *J* = 7.5 Hz), 3.75 (3H, s), 3.55 (1H, m), 3.74 (1H, t, *J* = 7.4 Hz), 3.29 (1H, t, *J* = 7.4 Hz), 2.84 (1H, dd, *J* = 13.5, 6.5 Hz), 2.67 (1H, dd, *J* = 13.5, 7.4 Hz). ^{13}C NMR (100 MHz, acetone-d_6, δ, ppm): 159.2 (C), 132.2 (C), 130.7 (CH), 130.7 (CH), 114.5 (CH), 114.5 (CH), 71.0 (CH_2), 60.5 (CH), 55.4 (CH_3), 39.1 (CH_2). ESIMS: 182.2 $[M + H]^+$.
